# Oxysterols Are Not Functionally Equivalent: Cell-Type- and Territory-Specific Responses in Human Models of Atherosclerosis

**DOI:** 10.3390/ijms27219629

**Published:** 2026-10-28

**Authors:** Damien Truffin, Flora Marchand, Mathias Chatelais

**Affiliations:** 1 Roquette Frères, Rue de la Haute Loge, 62136 Lestrem, France; 2 ProfileHIT, 3 les Glands, 44680 Sainte-Pazanne, France

**Keywords:** atherosclerosis, oxysterols, 7-ketocholesterol, 27-hydroxycholesterol, oxidized LDL, foam cells, vascular smooth muscle cells, macrophage polarization, cholesterol efflux, cyclodextrin

## Abstract

This study aimed to compare the biological activities of major oxysterols, including 7-ketocholesterol (7KC), 7α-hydroxycholesterol (7α-HC), 7β-hydroxycholesterol (7β-HC), and 27-hydroxycholesterol (27HC), as well as oxidized LDL (oxLDL), in complementary human models representative of the atherosclerotic microenvironment: monocyte-derived macrophages (MΦs), vascular endothelial cells (v-ECs), and vascular smooth muscle cells (v-SMCs) isolated from the carotid artery and abdominal aorta. Cellular responses were strongly dependent on both cell type and vascular territory. Among the oxysterols tested, 7KC exerted the strongest biological effects, markedly reducing cell viability, increasing oxidative stress and apoptosis, promoting endothelial dysfunction, and profoundly altering lipid homeostasis pathways. Spectral Nile Red analysis further revealed distinct patterns of neutral-lipid- and phospholipid-associated fluorescence, indicating oxysterol-specific remodeling of intracellular lipid compartments. In vascular smooth muscle cells, 7KC was associated with increased CD36 expression and reduced ABCA1/ABCG1-mediated cholesterol efflux, whereas macrophages responded predominantly through SREBP1- and NR1H4-associated metabolic reprogramming rather than enhanced lipid uptake. In contrast, oxLDL was the strongest inducer of neutral lipid accumulation and classical foam-cell formation in macrophages. Although 27HC increased intracellular 7KC levels, it induced comparatively limited effects on viability, oxidative stress, and macrophage functions. Moreover, oxLDL promoted a distinct macrophage activation state and altered polarization trajectories depending on the timing of exposure. These findings demonstrate that major oxysterols exert distinct and complementary biological effects across vascular and immune cell populations, highlighting their differential contribution to atherosclerotic plaque development and progression, and support the development of therapeutic approaches targeting multiple oxysterol species rather than a single oxidized cholesterol derivative.

## 1. Introduction

Atherosclerosis remains the leading cause of cardiovascular morbidity and mortality worldwide and is characterized by the progressive accumulation of lipids, inflammatory cells, and fibrous tissue within the arterial wall. Although hypercholesterolemia is a major causal factor, it is now well established that oxidative modifications of low-density lipoproteins (LDLs) play a central role in the initiation and progression of atherosclerotic lesions [1]. Among the products generated during cholesterol oxidation, oxysterols constitute a class of bioactive molecules involved in numerous pathological processes, including cardiovascular, neurodegenerative, ophthalmological, and inflammatory diseases, as well as cancers and sarcopenia. They are particularly implicated in the development and progression of atherosclerotic plaques [1,2,3].

**Figure 1:**
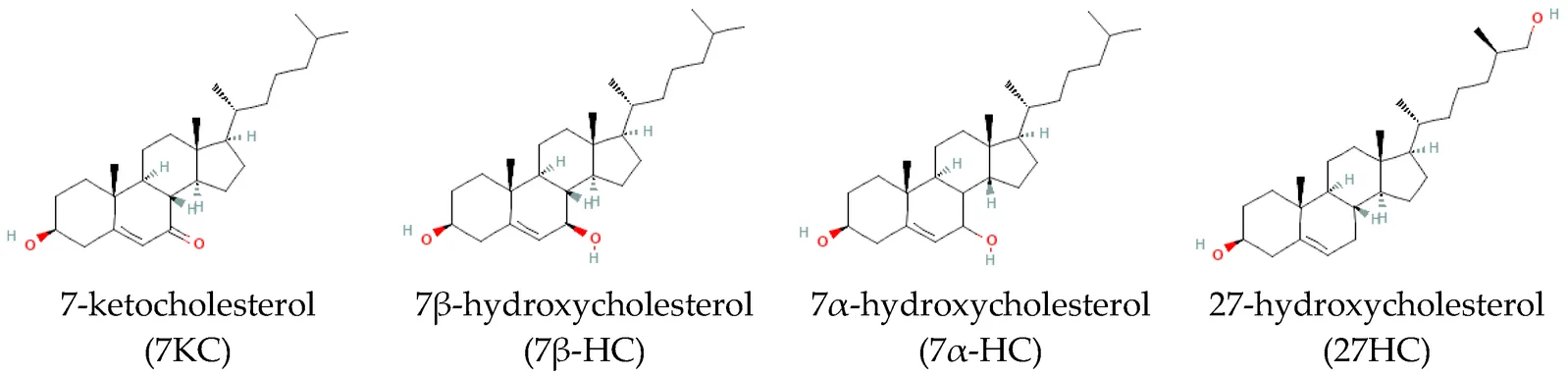
Chemical structures of oxycholesterols.

Oxysterols are oxidized derivatives of cholesterol generated either through enzymatic reactions, notably involving cytochrome P450 enzymes, or through non-enzymatic pathways associated with oxidative stress and lipid peroxidation [4,5,6,7]. These compounds may be produced locally within tissues, transported in circulating lipoproteins, or supplied through the diet via cholesterol-rich foods exposed to thermal processing or storage conditions that promote oxidation [1]. The most extensively studied oxysterols are 7-ketocholesterol (7KC), 7β-hydroxycholesterol (7β-HC), 7α-hydroxycholesterol (7α-HC), and 27-hydroxycholesterol (27HC) (Figure 1), whose biological activities vary according to their origin, concentration, and cellular environment [1,2,3,4].

Numerous studies have demonstrated that oxysterols accumulate in oxidized LDL as well as in human atherosclerotic lesions [1,4,8,9]. Their presence within plaques is associated with impaired vascular homeostasis involving endothelial cells, macrophages, and vascular smooth muscle cells. Oxysterols promote endothelial dysfunction, increased vascular permeability, the expression of adhesion molecules, and the recruitment of inflammatory cells, thereby contributing to the earliest stages of atherogenesis [1,4,10]. At the macrophage level, several oxysterols profoundly affect lipid metabolism, cholesterol efflux, and inflammatory polarization [11,12]. These effects favor the formation of foam cells, one of the hallmark features of early atherosclerotic lesions [13,14]. In parallel, oxysterols activate multiple inflammatory signaling pathways, including TLR4, NF-κB, and various pro-inflammatory cytokines, thereby establishing a close link between dysregulated sterol metabolism and chronic low-grade inflammation [1,2]. Furthermore, 7-KC and 7β-HC are particularly recognized for their cytotoxic properties including oxidative stress, inflammation and lipid homeostasis disruption, which can favor cell death induction [11]. These molecules induce reactive oxygen species production, mitochondrial dysfunction, endoplasmic reticulum stress, and various forms of cell death such as necrosis, necroptosis, apoptosis, oxiapoptophagy and ferroptosis [15,16,17]. Several studies have shown that these mechanisms contribute to plaque progression and instability. Apoptosis, impaired autophagy, and inflammation associated with these oxysterols are now considered promising therapeutic targets to limit cardiovascular complications [1,2]. Beyond their deleterious effects, certain oxysterols also exert important physiological signaling functions. In particular, 25-hydroxycholesterol (25HC) is a bioactive oxysterol generated mainly during inflammatory and infectious conditions that contributes to the regulation of lipid metabolism, immune responses, and several pathological processes, including atherosclerosis, neurological disorders, and cancer [18].

In particular, 25HC and 27HC regulate cholesterol homeostasis through activation of liver X receptors (LXRs) while exerting complex immunomodulatory activities [18,19,20]. This functional duality highlights the biological ambivalence of oxysterols and underscores the need for a better understanding of their respective roles in cardiovascular diseases [21,22]. Collectively, these findings support the concept that oxysterols should no longer be viewed merely as biomarkers of oxidative stress but rather as active mediators contributing to the pathophysiology of atherosclerosis and other age-related chronic diseases [22,23]. Consequently, the study of oxysterols has emerged as a major research field at the intersection of lipid metabolism, inflammation, and vascular biology. A deeper understanding of their mechanisms of action may lead to the identification of novel cardiovascular risk biomarkers and innovative therapeutic strategies designed to prevent the initiation, progression, and complications of atherosclerosis [1,2].

Moreover, growing evidence indicates that vascular smooth muscle cells are not merely passive components of the arterial wall but actively participate in atherosclerotic lesion development through phenotypic switching, inflammatory responses, and altered lipid handling. Indeed, vascular endothelial cells (v-ECs) and vascular smooth muscle cells (v-SMCs) can accumulate cholesterol and contribute to plaque remodeling, highlighting their importance as a cellular target for investigating the vascular effects of oxysterols [24].

Despite growing evidence supporting the involvement of oxysterols in atherosclerosis, important gaps remain in our understanding of their cell-specific effects and their contribution to vascular dysfunction. Most studies have focused on individual cell types, particularly macrophages, whereas atherosclerotic plaque development results from complex interactions among multiple cellular components of the vascular wall, including endothelial cells, vascular smooth muscle cells, and immune cells. Furthermore, oxysterols exert pleiotropic biological activities that differentially affect lipid homeostasis, inflammatory responses, cell survival, and stress-related signaling pathways depending on the cell type and biological context [11,12].

Therefore, the objective of the present study was to comprehensively characterize the biological effects of selected oxysterols and oxidized LDL in complementary human cellular models representative of the vascular microenvironment involved in atherosclerosis. The originality of this work lies in the combined use of macrophages and primary human vascular cells, including v-ECs and v-SMCs isolated from the carotid artery and abdominal aorta, two vascular territories associated with lipid-rich lesions [25]. By investigating the impact of oxysterols on lipid metabolism, inflammatory signaling, cellular stress responses, and phenotypic alterations across these distinct cell populations, this study aims to provide an integrated view of oxysterol-driven vascular dysfunction and to improve our understanding of their contribution to the initiation and progression of atherosclerotic disease. To address these questions, complementary human cellular models representative of the vascular microenvironment were investigated, including PBMC-derived macrophages, vascular endothelial cells, and vascular smooth muscle cells isolated from carotid and aortic territories. A comparative multiparametric approach was used to evaluate oxysterol-induced alterations in lipid accumulation, intracellular oxysterol content, lipid uptake and cholesterol efflux pathways, oxidative stress, apoptosis, endothelial dysfunction markers, phagocytic activity, and macrophage polarization. This strategy enabled the identification of cell-type- and vascular territory-specific responses to major oxysterols associated with atherosclerosis. To achieve this objective, a combination of flow cytometry, fluorescence microscopy, biochemical assays, and cytokine quantification approaches was used to characterize oxysterol uptake, lipid homeostasis, oxidative stress, cell death, inflammatory responses, and cell-specific functional alterations.

## 2. Results

### 2.1. Effects on Neutral Lipid and Phospholipid Accumulation

**Figure 2:**
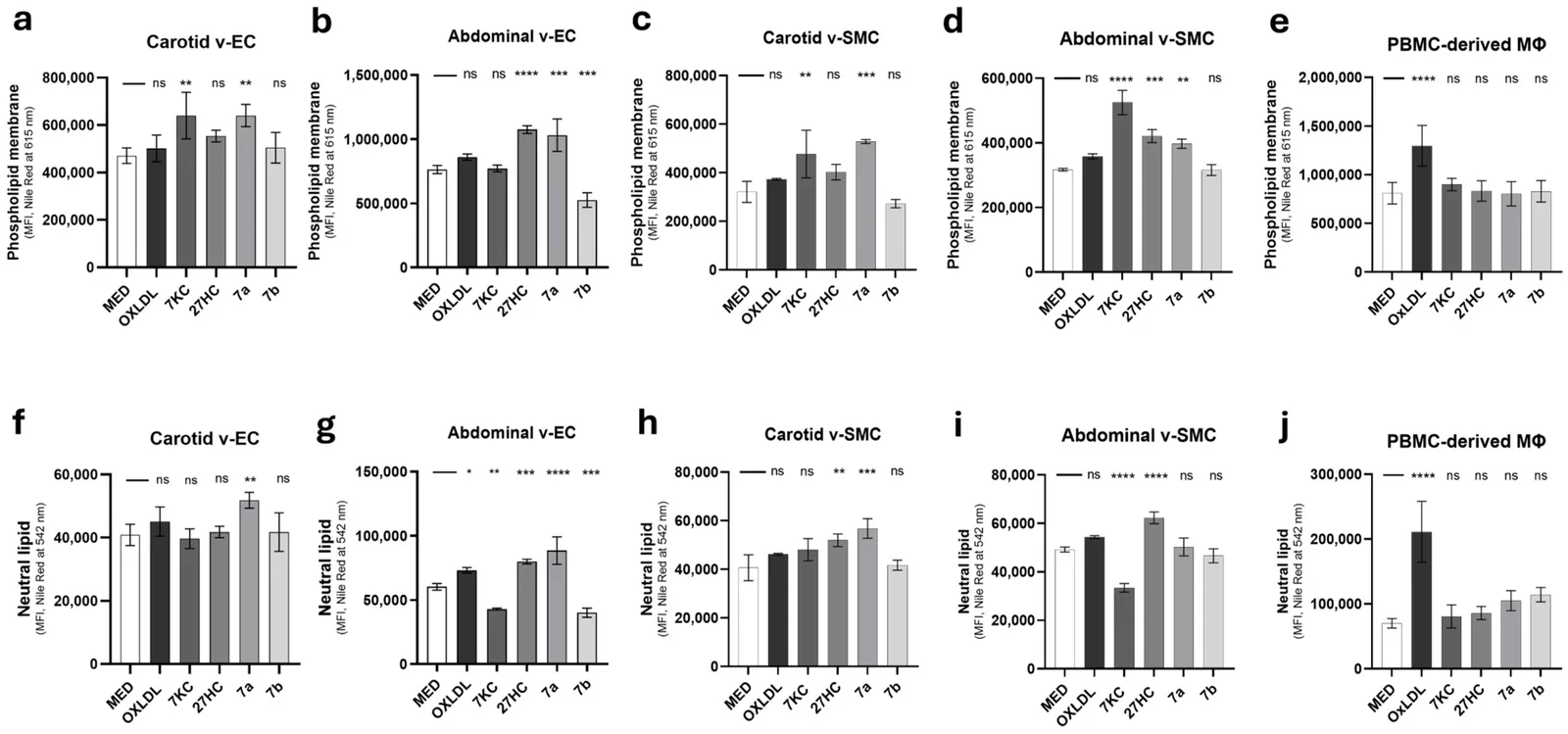
Phospholipid membrane and neutral lipid accumulation after 48 h of exposure to different oxysterols (oxLDL, 7KC, 27HC, 7a and 7b) in human carotid and abdominal vascular endothelial cells (v-ECs) (**a**,**b**,**f**,**g**), smooth muscle cells (v-SMCs) (**c**,**d**,**h**,**i**) and human PBMC-derived macrophages (PBMC-derived MΦs) (**e**–**j**). OxLDL tested at 30 µg/mL and oxysterols 7KC, 27HC, 7a and 7b at 40 µM. Detached cells stained 30 min with Nile Red 0.5 µM in PBS (Cat. No. 22190, AATBioquest, USA) at room temperature. A pool of 8 donors was used for carotid artery and abdominal aorta. Six individual donors were used for PBMC-derived macrophages. Data were collected on spectral flow cytometry, with phospholipid signal at 615 nm and neutral lipid signal at 542 nm (see Supplemental S1). Results are expressed in median fluorescence intensity ± SD. Each condition was performed in *n* = 3 technical replicates. A two-way ANOVA followed by Dunnett’s multiple comparison test was used to compare oxysterols with medium alone (MED), **** *p* < 0.0001, *** *p* < 0.001, ** *p* < 0.01, * *p* < 0.05, ns: non-significant.

To evaluate the impact of oxysterols associated with oxidized LDL on the major cell types involved in atherosclerosis, the effects of 7KC, oxLDL, 7α-HC (7a in the figures), 7β-HC (7b in the figures), and 27HC were investigated in macrophages (MΦs), vascular smooth muscle cells (v-SMCs), and vascular endothelial cells (v-ECs). Nile Red fluorescence was analyzed by spectral flow cytometry and deconvoluted into neutral-lipid-associated and phospholipid-associated components according to the spectral properties of the dye in order to better characterize oxysterol-induced lipid remodeling. Distinct patterns were observed depending on the oxysterol, cell type, and vascular territory (Figure 2). In carotid endothelial cells, 7α-HC induced the strongest increase (+26.86%) in neutral-lipid-associated fluorescence, whereas 7KC remained close to control levels. In abdominal endothelial cells, the highest neutral-lipid-associated signal was also observed following 7α-HC exposure (46.92%), while oxLDL and 27HC induced a moderate increase (21.26%, 32.64%), and both 7KC and 7β-HC were associated with reduced fluorescence compared with untreated controls (−28.83%, −33.50%). A similar heterogeneity was observed in vascular smooth muscle cells. In carotid v-SMCs, 7α-HC and 27HC generated the strongest neutral-lipid-associated signals (39.80%, 27.91%), whereas in abdominal v-SMCs, the largest increase was observed with 27HC (26.63%). In contrast, 7KC markedly reduced neutral-lipid-associated fluorescence in abdominal v-SMCs (−32.13%) while exerting no effect in carotid v-SMCs.

Analysis of the phospholipid-associated Nile Red signal revealed a distinct response pattern. In carotid endothelial cells, 7KC and 7α-HC markedly increased phospholipid-associated fluorescence (35.99%; 36.06%), with the strongest effects observed for 7α-HC. In abdominal endothelial cells, 27HC and 7α-HC generated the highest phospholipid-associated signals (40.78%, 34.99%), whereas 7KC remained close to the control, and 7β-HC reduced fluorescence (−31.17%). In carotid v-SMCs, phospholipid-associated fluorescence was strongly increased by 7α-HC and 7KC (64.44%, 48.48%). In abdominal v-SMCs, 7KC, 27HC and 7α-HC produced the greatest phospholipid-associated signals despite the reduction in neutral-lipid-associated fluorescence observed with 7KC (65.47%, 32.63%, 25.27%).

Macrophages displayed a markedly different profile (Figure 2). OxLDL induced the strongest increases in both neutral-lipid-associated and phospholipid-associated fluorescence (201.53% and 60.11%, respectively), consistent with robust intracellular lipid loading. In contrast, the other oxysterols did not significantly affect the response under the experimental conditions tested.

Overall, these findings indicate that oxysterols differentially remodel intracellular lipid compartments rather than uniformly promoting neutral lipid accumulation. Whereas 7α-HC and 27HC were the oxysterols most consistently associated with increased neutral-lipid-associated fluorescence in vascular cells, 7KC preferentially altered phospholipid-associated fluorescence in several vascular models, suggesting distinct mechanisms of lipid homeostasis deregulation according to both vascular territory and cell type.

### 2.2. Effects on Lipid Uptake Receptors and Cholesterol Efflux Pathways

Overall, the results revealed marked differences in the biological activity of the oxysterols tested. Among them, 7KC consistently induced the most pronounced cellular responses across all cell types examined. In macrophages, 7KC produced the largest changes in protein expression, with several parameters increasing more than six- to nine-fold compared with untreated controls. Similar trends were observed in vascular cells, suggesting a common susceptibility of the major cellular components of the arterial wall to this oxysterol. In contrast, oxidized LDL, 7α-hydroxycholesterol, 7β-hydroxycholesterol and 27-hydroxycholesterol generally induced more moderate effects. Although some cellular responses were significantly altered, their magnitude remained substantially lower than that observed with 7KC. These findings indicate that, despite partially overlapping mechanisms of action, oxysterols do not possess equivalent pathogenic potential. Collectively, our data identify 7KC as the most biologically active oxysterol under the present experimental conditions and suggest that it may be a major contributor to the cellular alterations associated with oxidized LDL.

**Figure 3:**
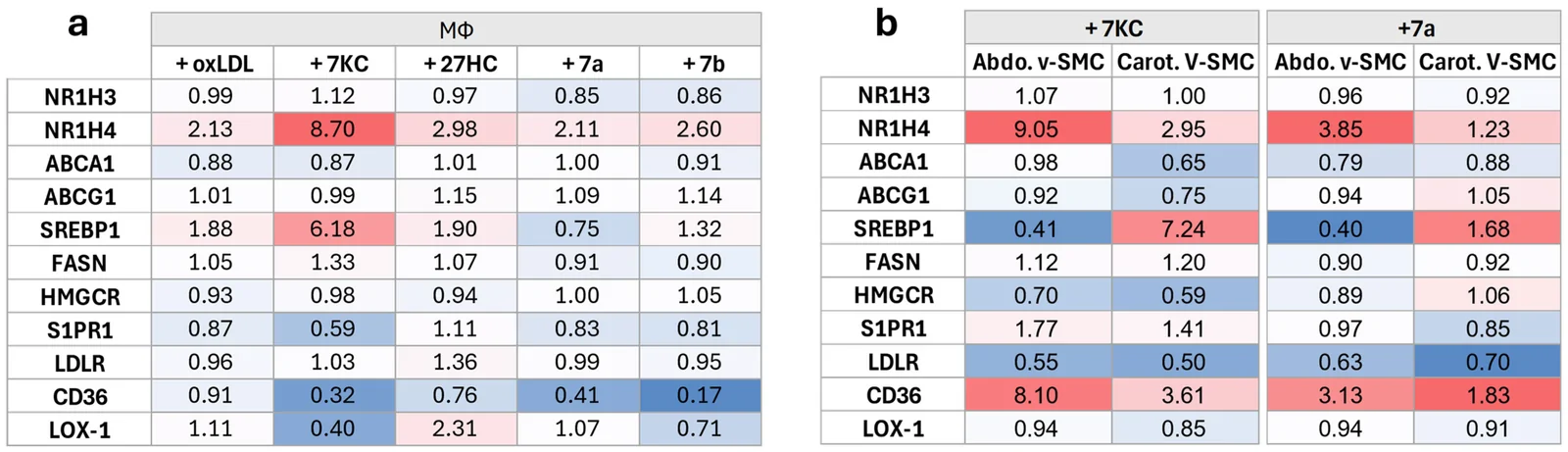
Human PBMC-derived macrophages (MΦs) were treated for 48 h with oxLDL [30 µg/mL] and oxysterols 7KC, 27HC, 7a and 7b [40 µM] (**a**). Vascular smooth muscle cells from carotid artery (Carot. v-SMCs) and abdominal aorta (Abdo. v-SMCs) were treated for 48 h with 7KC or 7a oxysterols [40 µM] (**b**). Detached and fixed cells (PFA 2%) were stained in PBS/2% FCS/2 mM EDTA/Triton 0.1% for lipid uptake receptors (LDLR, CD36, LOX1), cholesterol metabolism (NR1H3, NR1H4, SREBP1, FASN, HMGCR, S1PR1), and cholesterol efflux markers (ABCA1 and ABCG1); all references are available in Supplemental S2. Data were collected by spectral flow cytometry. HEATMAP view with numbers indicates fold increase (red) or decrease (blue) in expression compared to untreated control (medium alone). Each condition was performed in *n* = 3 technical replicates. A pool of 4 donors was used for each vascular smooth muscle cell territory. Three individual donors were used for PBMC-derived macrophages.

More specifically, exposure to 7KC induced distinct responses depending on the cellular model examined (Figure 3).

In macrophages, 7KC strongly increased the expression of NR1H4 (+8.7-fold) and SREBP1 (+6.18-fold), whereas the expression of the major scavenger receptors involved in the uptake of oxidized lipoproteins was reduced. In particular, CD36 and LOX-1 expression levels decreased to 0.32-fold and 0.40-fold of control levels, respectively. These findings suggest that the macrophage response to 7KC is not associated with enhanced uptake of extracellular lipids through classical scavenger receptor pathways. Notably, the overall response profiles obtained with the other oxysterols tested were qualitatively similar but considerably less pronounced.

Based on these observations, subsequent investigations in vascular smooth muscle cells focused primarily on 7KC and extended to 7α-HC according to foam-cell formation capability in v-SMCs (Figure 2c). Indeed, the proteomic analysis of lipid uptake and efflux pathways in v-SMCs was selectively directed toward the oxysterols that significantly promoted neutral lipid accumulation in these cells. Among the five conditions tested (Figure 2c,d), only 7KC and 7α-HC induced substantial foam-cell formation in v-SMCs from both vascular territories, whereas 7β-HC, 27HC and oxLDL did not elicit appreciable lipid loading in this compartment—consistent with the lower cytotoxic and lipogenic potential of 27HC reported in macrophages (Section 2.1). This criterion-based selection enabled a targeted investigation of the influx/efflux imbalance specifically underlying v-SMC–foam-cell conversion, rather than a dilution of the analysis across conditions with negligible lipogenic activity in this cell type.

In contrast to macrophages, vascular smooth muscle cells exhibited a proteomic profile consistent with enhanced lipid accumulation. CD36 expression was strongly induced in both aortic v-SMCs (+8.10-fold) and carotid v-SMCs (+3.61-fold). This increase was accompanied by a reduction in the expression of the cholesterol efflux transporters ABCA1 and ABCG1, as well as decreased LDLR expression. Moreover, SREBP1 expression was markedly upregulated in carotid v-SMCs (+7.24-fold), whereas an opposite response was observed in aortic v-SMCs (0.41-fold).

Notably, although 7α-HC also promoted foam-cell formation in both carotid and abdominal v-SMCs (Figure 2c,d), it did not reproduce the receptor/transporter profile induced by 7KC, suggesting that alternative intracellular pathways—independent of the CD36/ABCA1 axis—may contribute to lipid accumulation in v-SMCs exposed to this oxysterol. These results suggest that oxysterol-sensitized smooth muscle cells can lead to foam-cell formation, depending on specific internal mechanisms adapted to oxysterol qualities.

### 2.3. Intracellular 7KC Accumulation Predicts Oxysterol-Induced Cytotoxicity Evaluated by Cell Counting

**Figure 4:**
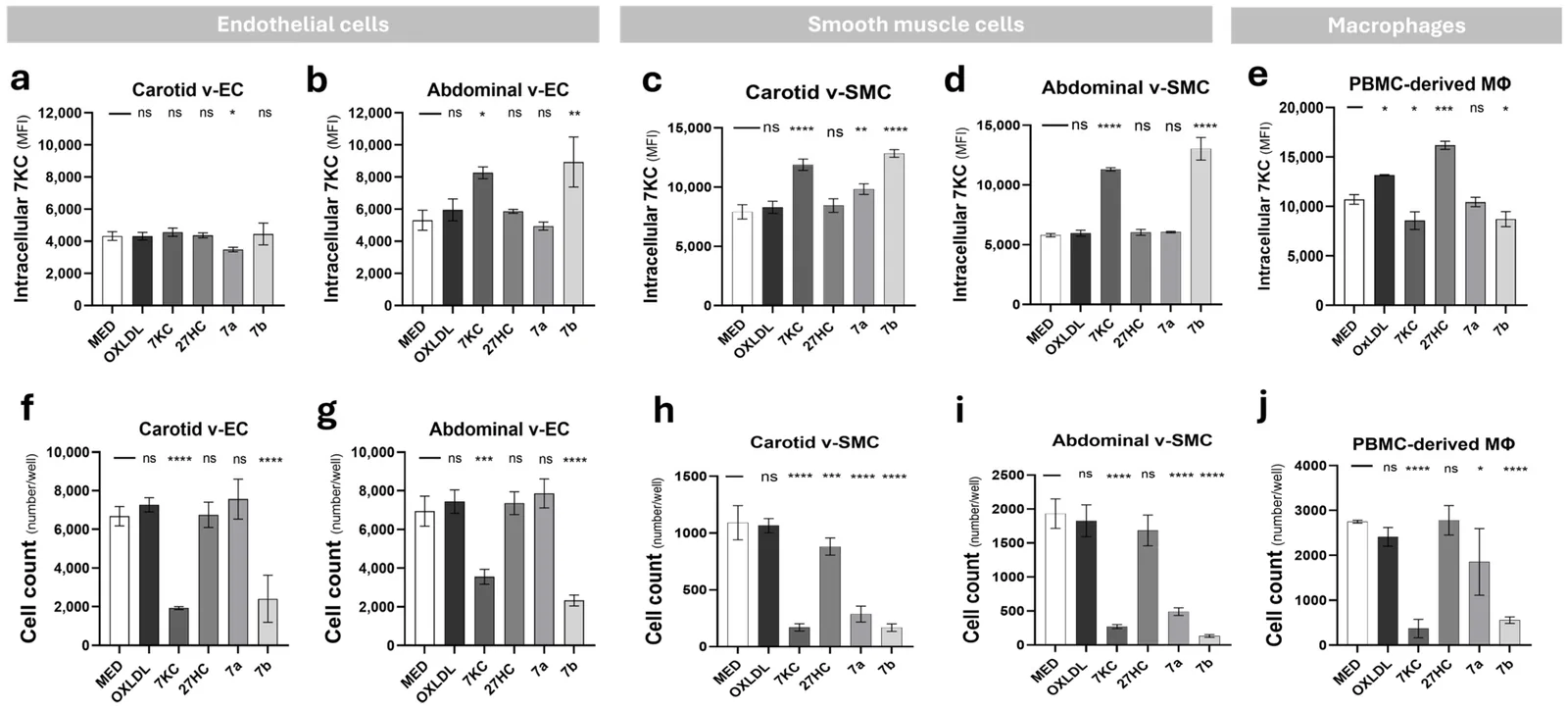
Intracellular 7KC accumulation after a 48 h exposure to different oxysterols (oxLDL, 7KC, 27HC, 7a and 7b) in human carotid and abdominal vascular endothelial cells (v-ECs) (**a**,**b**), smooth muscle cells (v-SMCs) (**c**,**d**), and human PBMC-derived macrophages (PBMC-derived MΦs) (**e**). OxLDL tested at 30 µg/mL and oxysterols 7KC, 27HC, 7a and 7b at 40 µM. Detached and fixed cells (PFA 2%) were stained in PBS/2% FCS/2 mM EDTA/Triton 0.1% for anti-human 7KC (Cat. No. MA5-45793, clone 7E1; Invitrogen, Carlsbad, CA, USA) for 30 min on ice. Cell toxicity was addressed using the absolute counting per well (cell count) in the different models (**f**–**j**). A pool of eight donors was used for carotid artery and abdominal aorta. Three individual donors were used for PBMC-derived macrophages. Data were obtained by spectral flow cytometry and expressed as median fluorescence intensity or absolute cell count per well ± SD. Each condition was performed in *n* = 3 technical replicates. A two-way ANOVA followed by Dunnett’s multiple comparison test was used to compare oxysterols with medium alone (MED), **** *p* < 0.0001, *** *p* < 0.001, ** *p* < 0.01, * *p* < 0.05, ns: non-significant.

These proteomic differences were consistent with measurements of the intracellular 7KC content (Figure 4). Indeed, intracellular accumulation of 7KC was observed in both v-SMCs and aortic endothelial cells following oxysterol exposure, whereas no increase was detected in carotid endothelial cells. In contrast, macrophages displayed reduced intracellular 7KC levels despite continuous exposure to both 7KC and 7β-HC (7b). Conversely, exposure to oxLDL and, surprisingly, to 27HC led to a significant increase in intracellular 7KC accumulation.

If we look at the relationship between the number of cells and intracellular 7KC, intracellular 7KC accumulation does not fully predict oxysterol-induced loss of viability.

In carotid endothelial cells, exposure to 7KC markedly reduced the cell number (71.18% decrease vs. control), while intracellular 7KC levels remained largely unchanged (5.46% change vs. control). A similar pattern was observed following 7β-HC treatment, which decreased cell number by approximately 64.0% despite only limited alterations in the intracellular 7KC content. These findings indicate that substantial endothelial toxicity can occur in the absence of significant intracellular 7KC accumulation.

By contrast, in aortic endothelial cells, 7KC exposure increased intracellular 7KC levels by 1.56-fold and was associated with a 48.82% reduction in cell number. Similarly, 7β-HC treatment induced both intracellular 7KC accumulation (1.68-fold increase) and a marked decrease in viability (66.47% decrease). In these cells, intracellular 7KC accumulation and cell viability appeared to be closely associated.

A different profile was observed in macrophages. Exposure to oxLDL increased intracellular 7KC levels by approximately 1.23-fold, while the cell number decreased by only 12.32%. Unexpectedly, 27HC also induced a marked increase in intracellular 7KC (1.51-fold) without a proportional reduction in viability. These observations suggest that macrophages can tolerate substantial intracellular oxysterol accumulation without undergoing the pronounced cytotoxic responses observed in endothelial cells.

Overall, the relationship between the intracellular 7KC content and cell viability was highly dependent on cellular context. While intracellular 7KC accumulation was associated with cell viability in certain cell types, particularly aortic endothelial cells, it was neither necessary nor sufficient to predict loss of viability across all experimental models.

### 2.4. Intracellular 27-Hydroxycholesterol (27HC)

Similar to the effects observed following 27HC exposure, a significant increase in intracellular 7KC levels was detected. This finding was unexpected, as 27HC and 7KC are generated through distinct biosynthetic pathways, and 7KC is not considered a direct metabolite of 27HC. To investigate whether the observed increase in intracellular 7KC could result from a cellular conversion of 27HC, intracellular 27HC concentrations were quantified and compared with the corresponding 7KC levels in exposed cells.

**Figure 5:**
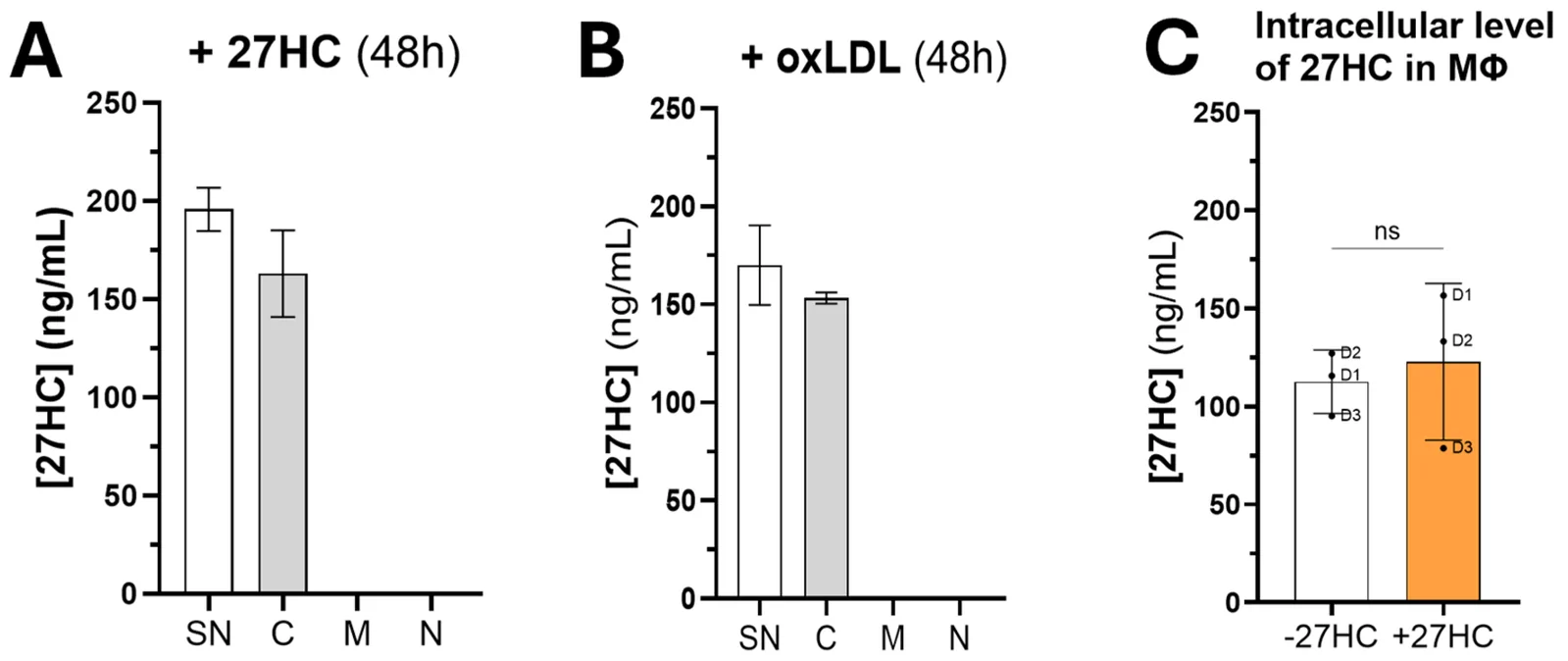
27HC levels in human PBMC-derived macrophages measured by ELISA after a 48 h exposure to different oxysterols (oxLDL and 27HC). Isolation of membrane, cytoplasm and nucleus compartments was achieved using a commercial cell fractionation kit (Cat. No. 42193S; Cell Signaling Technology, Danvers, MA, USA) according to the manufacturer’s instructions. 27HC level expressed in pg/mL in the different fractions (SN: supernatant, C: cytoplasm, M: membrane, N: nucleus) following a 48 h treatment with 40 µM 27HC (**A**) or 30 µg/mL oxLDL (**B**). No detection was observed in Membrane and Nucleus compartments. A comparative approach of 27HC levels by ELISA (Cat. No. MBS1608736; Mybiosource, San Diego, CA, USA) after a 48 h exposure to 27HC was proposed in 3 individuals’ human PBMC-derived macrophages (**C**). Each condition was performed in *n* = 3 technical replicates. Results are presented as mean ± SD. Mann–Whitney *t*-test, ns: non-significant.

The results presented in Figure 5 show that exposure to 27HC does not lead to an increase in intracellular 27HC levels. Therefore, the increase in intracellular 7KC observed following 27HC exposure is likely associated with a distinct mechanism of action.

### 2.5. Vascular Effects of Oxysterols on Endothelial Dysfunction Markers

To determine whether the vascular effects observed with 7KC were specific to this oxysterol or could also be induced by other oxysterols, we included 7α-HC (7a), which exhibited weaker effects on cell viability and lipid receptor regulation in our cellular models. This comparative approach allowed us to evaluate whether endothelial alterations were a common consequence of oxysterol exposure or predominantly associated with the more deleterious properties of 7KC.

**Figure 6:**
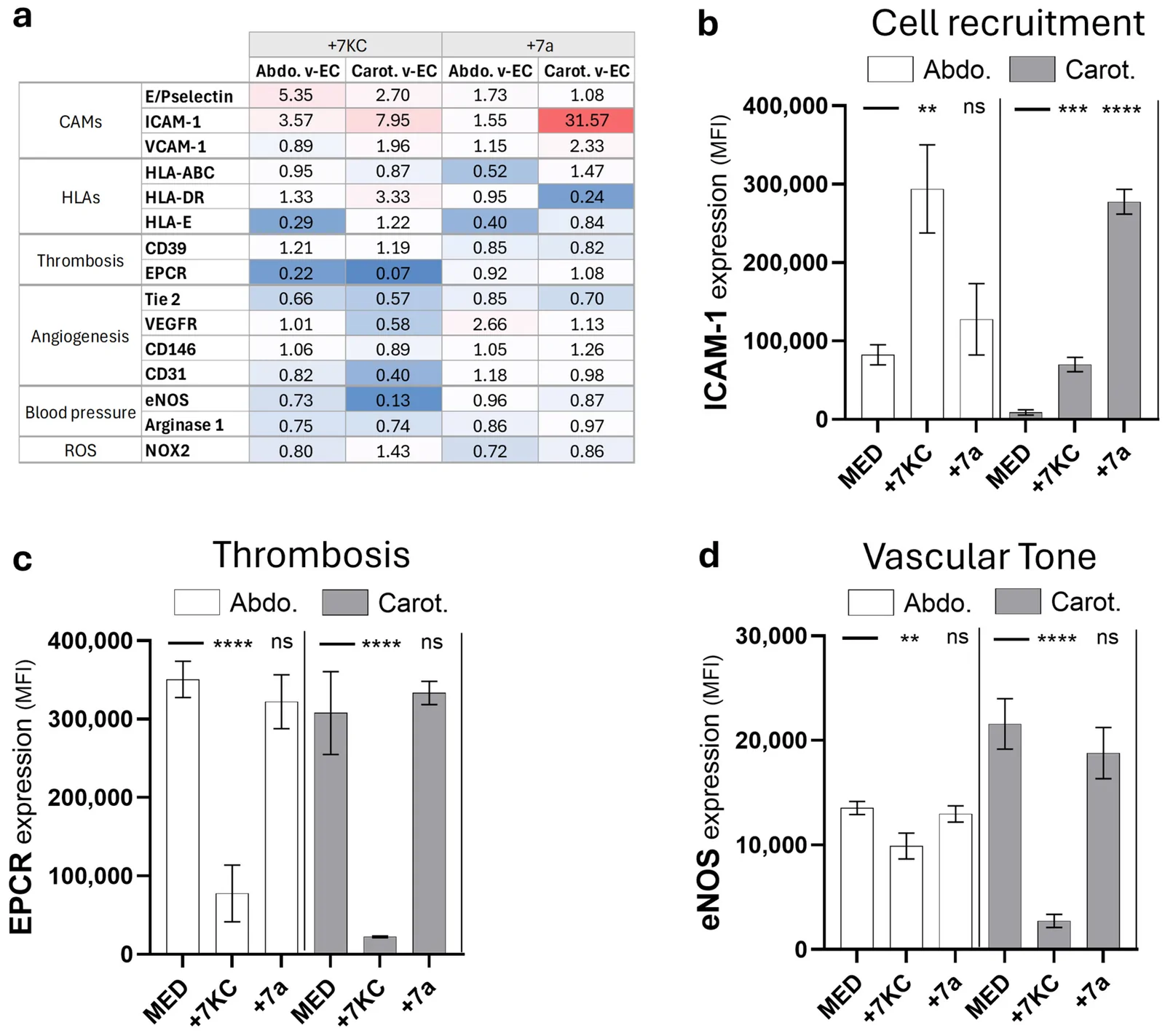
Vascular endothelial cells from carotid artery (Carot. v-ECs) and abdominal aorta (Abdo. v-ECs) were treated for 48 h with 7KC or 7a oxysterols [40 µM] (**a**). Detached cells (Accutase, Corning) were stained in PBS/2% FCS/2 mM EDTA for cell adhesion molecules (E/Pselectin, ICAM-1, VCAM-1), human leukocyte antigens (HLA-ABC, HLA-E, HLA-DR), thrombosis (CD39, EPCR) and angiogenic markers (Tie2, VEGFR2, CD146, CD31). Detached and fixed cells (PFA 2%) were stained in PBS/2% FCS/2 mM EDTA/Triton 0.1% for lipid blood pressure (eNOS, Arginase 1) and reactive oxygen species (ROS) enzymes (NOX2); all references are available in Supplemental S2. HEATMAP view with numbers indicates fold increase (red) or decrease (blue) in expression compared to untreated control (medium alone). Each condition was performed in *n* = 3 technical replicates. A pool of eight donors was used for each vascular territory. An overview of ICAM-1 (**b**), EPCR (**c**) and eNOS (**d**) expression in both vascular territories following a 48 h 7KC and 7a exposure compared to untreated control. Results are presented in bar graphs as mean ± SD. Ordinary one-way ANOVA followed by Dunnett’s multiple comparison test was used to compare oxysterols with medium alone (MED), **** *p* < 0.0001, *** *p* < 0.001, ** *p* < 0.01, ns: non-significant.

As illustrated in Figure 6, both oxysterols markedly modulated the expression of vascular markers involved in leukocyte recruitment, thrombosis, and vascular homeostasis.

Among the adhesion molecules examined, ICAM-1 expression levels were strongly upregulated by both compounds, although the pattern differed according to the vascular bed. In aortic endothelial cells, 7KC increased ICAM-1 expression from approximately 80,000 to 290,000 arbitrary units, whereas in arterial endothelial cells, the strongest response was observed following 7α-HC exposure, with ICAM-1 levels increasing from approximately 70,000 to 280,000 arbitrary units (Figure 6b). These findings suggest that oxysterols can directly promote an activated endothelial phenotype favoring leukocyte adhesion and monocyte recruitment, two key early events in atherogenesis.

In addition to their effects on endothelial activation and vascular tone, the two tested oxysterols altered the expression of markers associated with antigen presentation and thrombosis. Within the HLA family, 7KC induced a marked upregulation of specific HLA molecules, particularly in arterial endothelial cells, where expression levels of HLA-DR increased by approximately 3-fold, and there was a 3-fold decrease in HLA-E in the aorta. In contrast, 7α-HC produced more modest and variable responses, including decreases in the HLA components. Regarding thrombosis-related markers, distinct oxysterol-dependent effects were observed. While most markers exhibited limited changes, exposure to 7KC resulted in a pronounced reduction in EPCR expression in both aortic and arterial endothelial cells (Figure 6c).

Moreover, oxysterols also modulated the expression of angiogenesis-associated markers. Exposure to 7KC resulted in a global downregulation of several angiogenic factors in both vascular models, with expression levels reduced to approximately 40–60% of control values for some targets, especially for Tie2, VEGFR (for artery), and CD31. These findings suggest that 7KC may impair endothelial processes involved in vascular remodeling and neovascular formation. In contrast, 7α-HC exerted more limited effects on angiogenesis-related proteins. While most markers remained close to control levels, one angiogenic marker, VEGFR, was markedly induced following 7α-hydroxycholesterol exposure, indicating that this oxysterol does not reproduce the broad anti-angiogenic profile observed with 7KC (Figure 6a).

Furthermore, alterations in vascular tone regulation were also observed. Exposure to 7KC induced a marked reduction in endothelial nitric oxide synthase (eNOS), and, to a lesser extent in Arginase 1 expression levels in both endothelial models, with the most pronounced effect detected in arterial endothelial cells (0.87-fold decrease). These effects were considerably more pronounced for 7KC than for 7α-HC, suggesting that the severity of endothelial dysfunction correlates with the intrinsic biological activity of the oxysterol (Figure 6d).

Finally, NOX2 expression, an NADPH oxidase linked to reactive oxygen species and nitric oxide production in endothelial cells, was modestly upregulated in arterial endothelial cells exposed to 7KC (up to 1.43-fold relative to control), whereas exposure to 7α-HC did not markedly affect NOX2 expression (0.72–0.86-fold).

Because 7KC was identified as the most potent inducer of oxidative stress and cell viability, whereas 27HC strongly modulated intracellular sterol homeostasis without causing marked cell death, these two oxysterols were selected for further phagocytosis studies. The inclusion of oxLDL provided a clinically relevant comparator representative of the atherosclerotic microenvironment.

### 2.6. Effects of Oxysterols on Oxidative Stress, Apoptosis, and Phagocytic Activity in Macrophages

**Figure 7:**
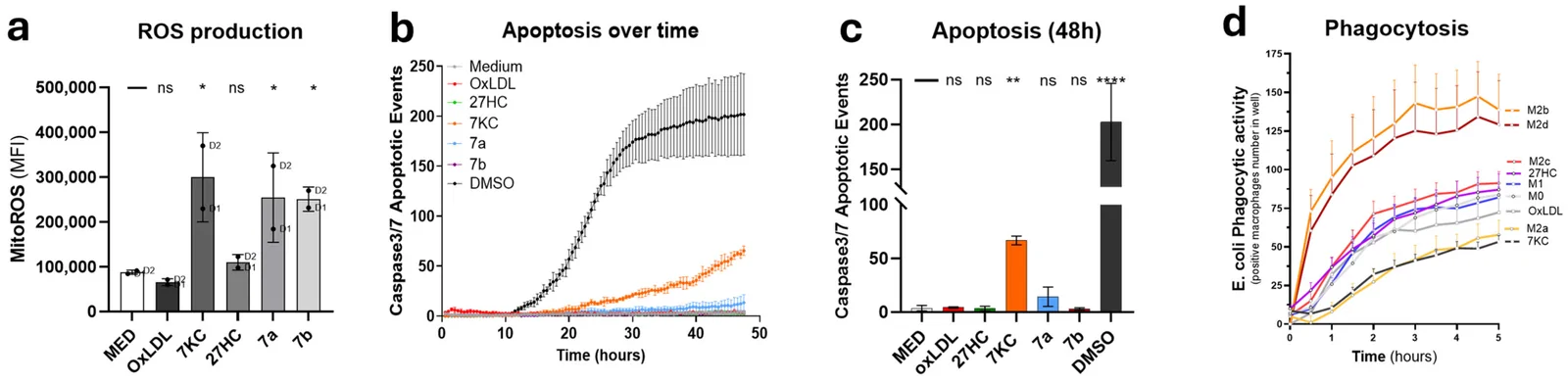
Human PBMC-derived macrophages (MΦs) treated for 48 h with oxLDL [30 µg/mL] or oxysterols 7KC, 27HC, 7a and 7b [40 µM], monitored for mitochondrial ROS production (MitoROS 580, AATbioquest) at 24 h in two individual donors (**a**). The onset of apoptotic events (CellEvent™ Caspase-3/7 Detection Reagents, Cat. No. C10432, Invitrogen, USA) obtained after 48 h by video microscopy (**b**) and illustrated at a 48 h endpoint (**c**) in three individual donors. Results are presented as mean ± SD. Phagocytosis of *E. coli* bioparticles (pHrodo Green e. coli BioParticles, Cat. No. P35366, Invitrogen, USA) monitored over time in polarized macrophages (**d**). M0 (medium), M1 (INFg; 20 ng/mL), M2a (IL4, 5 ng/mL), M2b (LPS, 100 ng/mL + poly I:C, 200 ng/mL), M2c (IL10, 20 ng/mL), M2d (LPS, 100 ng/mL + IL6, 50 ng/mL), oxLDL (30 µg/mL), 27HC (40 µM) and 7KC (40 µM). Signal acquired on a Muvicyte imager (Muvicyte, PerkinElmer, Waltham, MA, USA), with the image captured every 30 min for 5 h; results are presented as mean ± SEM. Each condition was performed with *n* = three technical replicates. Two-way ANOVA followed by Dunnett’s multiple comparison test was used to compare oxysterols with medium alone (MED), **** *p* < 0.0001, ** *p* < 0.01, * *p* < 0.05, ns: non-significant.

To further evaluate the cellular effects of oxysterol exposure, ROS production, apoptosis, and phagocytic activity were assessed in macrophages. Exposure to 7KC, 7α-HC and 7β-HC markedly increased intracellular ROS generation (241.77%, 190.16% and 185.96%, respectively) compared with control cells and oxLDL and 27HC (Figure 7a). This increase was accompanied by a significant induction of apoptosis for 7KC, consistent with the pronounced cytotoxic effects observed in response to 7KC. In contrast, 7α-HC, 7β-HC, oxLDL and 27HC elicited weaker effects on apoptotic responses (Figure 7b,c).

Macrophage phagocytic activity differed markedly according to polarization status and oxysterol exposure (Figure 7d). Among the macrophage subtypes, M2b and M2d exhibited the highest phagocytic activity throughout the experiment, reaching values close to 140–150 positive events after 4–5 h. M2c macrophages displayed an intermediate phenotype, with phagocytic activity consistently higher than that observed in M0 and M1 macrophages. Exposure to 27HC resulted in a phagocytic profile similar to that of M2c macrophages, with a progressive increase in uptake over time.

In contrast, macrophages exposed to oxLDL exhibited a reduced phagocytic capacity. Although phagocytosis initially increased during the first hours of incubation, a clear plateau followed by a decline in activity was observed after approximately 3 h. A similar impairment was observed following exposure to 7KC. Indeed, 7KC-treated macrophages showed one of the lowest phagocytic activities among all experimental conditions, closely resembling the M2a phenotype. After a modest initial increase, phagocytic activity remained markedly reduced compared with M0, M1, M2c, and 27HC-treated cells throughout the time course.

Overall, these results identify M2b and M2d macrophages as highly phagocytic phenotypes, whereas exposure to oxLDL or 7KC is associated with a substantial reduction in macrophage phagocytic function.

### 2.7. Effects of Oxysterols on Macrophage Polarization

Based on the marked cytotoxic effects induced by 7KC, including increased ROS production, apoptosis, and reduced cell viability, this oxysterol was not further investigated in macrophage polarization experiments. Under these conditions, changes in polarization markers could have reflected cell death rather than genuine macrophage reprogramming. Therefore, subsequent analyses focused on oxLDL and 27HC, two atherogenic stimuli exerting more moderate effects on cell viability while remaining highly relevant to the pathophysiology of atherosclerosis. In addition, 7α-HC and 7β-HC were not selected for further polarization studies because their biological effects were considerably weaker than those observed with 27HC. As the objective was to investigate macrophage polarization induced by clinically relevant and biologically active atherogenic stimuli, subsequent experiments were restricted to oxLDL and 27HC, which provided the most relevant models for investigating oxysterol-mediated macrophage reprogramming.

**Figure 8:**
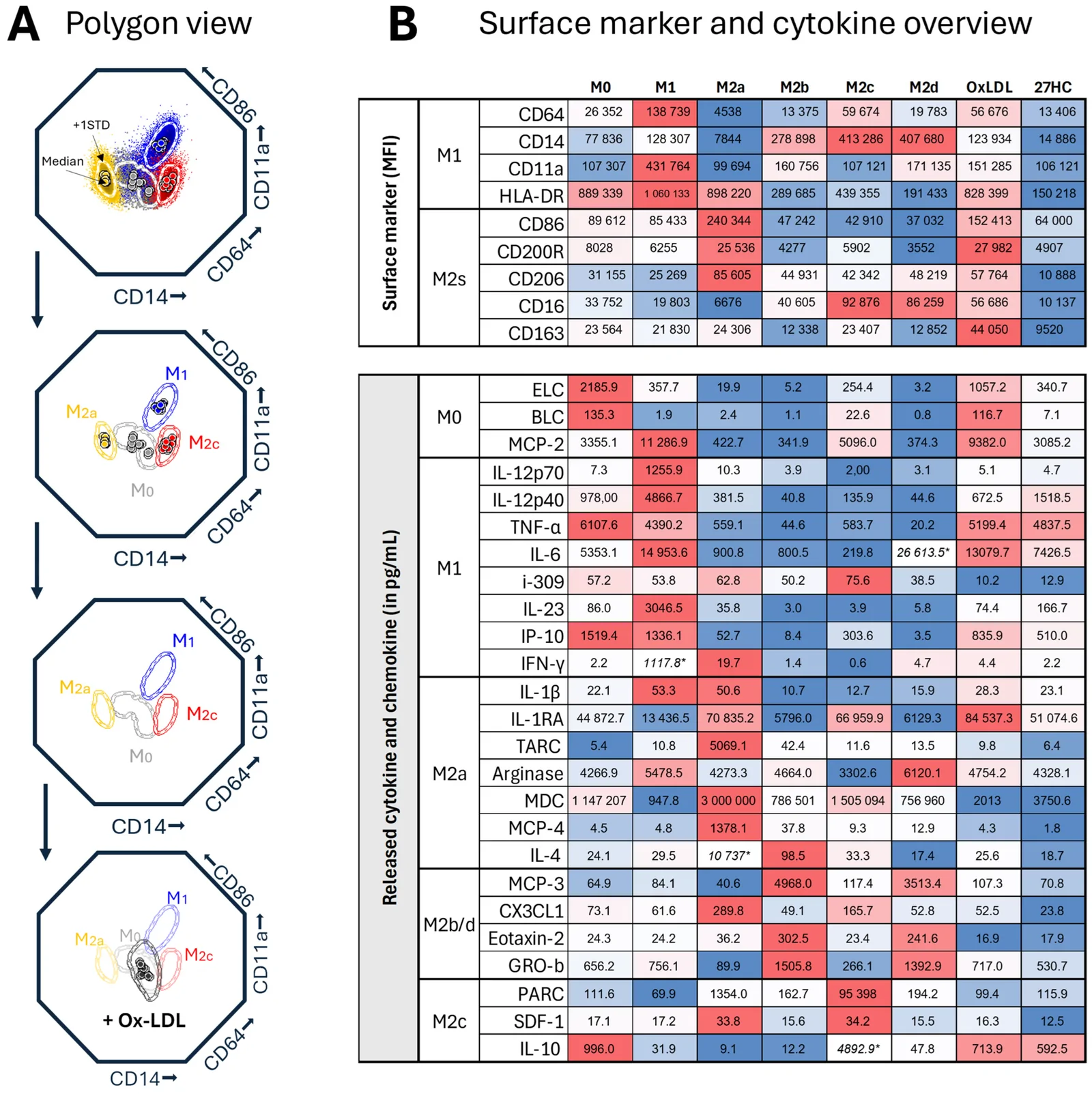
Flowcytometry phenotype of polarized macrophages (M0, M1, M2a and M2c), according to the 4 discriminant markers CD64, CD14, CD11a and CD86 (**A**). Clustering of six individual donors illustrated in a unique polygon plot representation using simultaneous combination of the four parameters. Each individual donor median is represented by a circular dot; the standard deviation of the group is represented by the dotted line and is used to compare with oxLDL treatment alone. A non-exhaustive comparative phenotype expression is proposed between polarized macrophages and oxysterol-treated macrophages (**B**). IFN-γ, IL-10, IL-4, and IL-6 (in italics and marked with an * in the table) were used in experimental conditions to stimulate the cells in order to polarize in M1, M2a, etc. M0 (medium), M1 (INFg; 20 ng/mL), M2a (IL4, 5 ng/mL), M2b (LPS, 100 ng/mL + poly I:C, 200 ng/mL), M2c (IL10, 20 ng/mL), M2d (LPS, 100 ng/mL + IL6, 50 ng/mL), oxLDL (30 µg/mL) and 27HC (40 µM) were measured for expression of surface markers (CD64, CD14, CD11a, HLADR, CD86, CD200R, CD206, CD163) and 25 cytokines released into the supernatant following a 24 h LPS boost (100 ng/mL). Cytokines expressed in pg/mL were assigned to macrophage subpopulations according to the intensity of expression. The cytokines ELC, BLC and MCP-2 are assigned to M0 population; IL-12p70, IL-12p40, TNF-α, IL-6, i-309, IL-23, IP-10 and IFN-γ refer to M1; IL-1β, IL-1RA, TARC, arginase, MDC, MCP-4, IL-4 are allocated to M2a; MCP-3, CX3CL1, Eotaxin-2, and GRO-b indicate M2b/d; and PARC, SDF-1, and IL-10 are assigned to M2c phenotype. Each analyte was dosed in *n* = 2 technical replicates from six individual donors. HEATMAP view with numbers indicates fold increase (red) or decrease (blue) in expression compared to untreated control (medium alone).

Phenotypic characterization confirmed the successful generation of distinct macrophage activation states (Figure 8). M1 macrophages were characterized by a marked increase in CD64, CD11a, and HLA-DR expression, whereas the expression of CD14, CD200R, CD86, CD206 and CD163 remained low. In contrast, M2a macrophages exhibited the most pronounced alternative activation profile, with significant upregulation of CD200R, CD86 and CD206 associated with reduced CD64, CD14, CD11a and CD16 expression.

M2b, M2c and M2d macrophages shared several common features, notably a strong increase in CD14 expression and moderate elevation in CD206 while maintaining low expression of CD200R and CD86. However, M2c differed by exhibiting increased CD64 expression, whereas M2b and M2d displayed reduced CD64 levels.

OxLDL exposure induced a distinct macrophage phenotype marked by the upregulation of nearly all surface markers examined. CD200R, CD86, and CD163 were the most strongly induced, while CD64 and CD206 showed a more moderate increase. This profile differed from both classical M1 and canonical M2 phenotypes, suggesting the emergence of a specific lipid-associated activation state.

In contrast, exposure to 27HC resulted in a global reduction of most phenotypic markers, including CD14, CD206 and CD163, indicating a weakly activated phenotype that did not closely resemble any of the reference polarization states.

Clustering macrophage populations in flow cytometry remains a challenging step. Based on differential expression, we successfully discriminated M0 from M1, M2a, M2c with a Polygon Plot view, where the markers CD14/CD64/CD11a/CD86 are expressed together in the same figure (Figure 8A). This new mode of representation allows us to drive results according to phenotypic variability obtained with multiple donors for the main M1/M2 populations, summarized in one unique figure and used as a stamp for downstream comparison with other conditions. Even if this representation gives clear insight into classical M0/M1/M2 polarization, oxLDL treatment did not support a clear distinct phenotype from conventional macrophages. As a further analysis, we investigated cytokine/chemokine secretion. Cytokine release by macrophages is a key driver of immune response in vitro and in vivo. To discriminate the oxLDL signature from conventional M1/M2 macrophages, we measured cytokine expression in the supernatant. A panel of 25 cytokines were detected in addition to surface markers to highlight specificities; the results are depicted in the heatmap view (Figure 8B).

As expected, M1 macrophages displayed the strongest pro-inflammatory cytokine signature, with marked increases in IL-6, TNFa, IL-12p40, IL-12p70, IL-1β, IL-23 and IP10 associated with decreased expression of IL-1RA.

M2a macrophages exhibited a mixed cytokine profile, characterized by reduced production of IL-6, TNFa, IL-12p40, IL23 and IP10, along with increased expression of IL-1RA, TARC, and MCP4. Despite this anti-inflammatory tendency, IL-1β levels remained elevated.

M2b and M2c macrophages showed a generally attenuated inflammatory profile, with marked reduced expression of IL-6 and IL-12p40. M2c differed from M2b by displaying a strong increase in PARC, SDF1 and IL-1RA expression.

M2d macrophages exhibited a unique cytokine pattern characterized by strong arginase, MCP3, eotaxin-2 and GroB production associated with reduced expression of IL-23, IL-12p40, IL-1RA, and IP10, while IL-1β levels remained unchanged.

By comparison, 27HC had only limited effects on cytokine production. Most cytokines remained unchanged, with only slight increases detected in the expression of IL-6, IL-12p40, and TNFa and a minimal reduction in IL-1RA.

Exposure to oxLDL induced a predominantly inflammatory response, with marked increases in the expression levels of TNFa, IL6 and IL-1RA, whereas IL-1β and IL-23 remained largely unchanged. Interestingly, oxLDL treatment alone is not sufficient to induce a clear polarization toward the M1 phenotype, and M2 cytokine co-expression (IL1R1A, Arginase and IL10) with M0 cytokines (ELC, BLC) maintains this ambiguous phenotype. These intriguing results prompted us to investigate the role of oxLDL before and after polarization to better understand whether oxysterols can determine the pro- or anti-inflammatory phenotype depending on when they accumulate in the atherosclerotic plaque.

**Figure 9:**
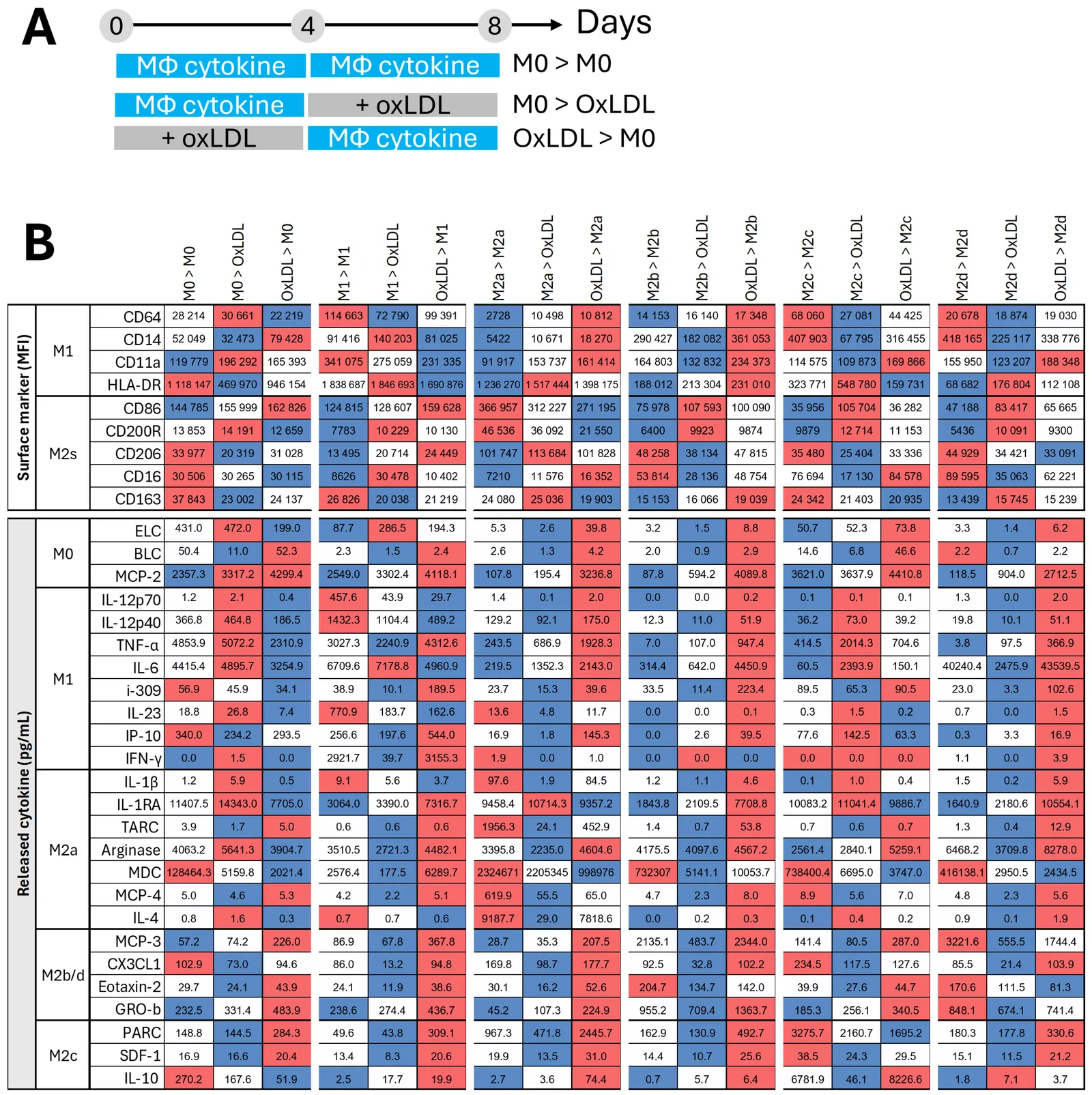
Protocol used to study oxLDL’s role before and after macrophage polarization (**A**). After plating, cells were treated with medium or cytokine or oxLDL; on day 4, medium was changed to medium, cytokine or oxLDL depending on the initial treatment. M0 > M0 represents macrophages incubated for 8 days in medium; oxLDL > M0 represents macrophages incubated for 4 days in oxLDL + 4 days in medium; M0 > oxLDL represents macrophages incubated for 4 days in medium and challenged 4 additional days with oxLDL. Results of phenotypic surface markers expressed in MFI (CD64, CD14, CD11a, HLADR, CD86, CD200R, CD206, CD163) and cytokines released into the supernatant expressed in pg/mL are summarized in the table for each polarized condition (**B**). Red and blue indicate higher or lower values in a set of three data compared (i.e., M0 > M0 versus oxLDL > M0 versus M0 > oxLDL). M0 (medium alone), M1 (INFg; 20 ng/mL), M2a (IL4, 5 ng/mL), M2b (LPS, 100 ng/mL + poly I:C, 200 ng/mL), M2c (IL10, 20 ng/mL), M2d (LPS, 100 ng/mL + IL6, 50 ng/mL), oxLDL (30 µg/mL). Each analyte and phenotypic marker was measured in *n* = 2 technical replicates from four individual donors.

The observation that oxysterols differentially modulate macrophage surface markers raised the question of whether this modulation extends to a reprogramming of the polarization process itself and whether the outcome depends on the timing of exposure. Among the conditions tested, oxLDL emerged as the strongest modulator of macrophage polarization, inducing pronounced increases in CD200R, CD86, and CD163 expression, while 27HC produced only limited phenotypic changes. We therefore focused subsequent investigations on oxLDL and challenged macrophages either before or after polarization (Figure 9A).

Exposure to oxLDL before polarization (oxLDL > MΦ, Figure 9B) changes the phenotype of M2a, M2b and M2c macrophages. These populations are the most sensitive to oxysterols, losing their alternative phenotype by exhibiting a pro-inflammatory (IL12-p40, IL6, TNFa) or dysregulated signature (MCP4, PARC, GroB), confirming the ability of oxLDL to reprogram macrophages depending on the local context. These findings suggest that oxLDL can interfere with natural macrophage programming pathways. Conversely, M1-polarized macrophages exhibit a relative reduction in certain inflammatory markers (IL-12p40, IL6, IL23) in the presence of oxLDL, accompanied by an increase in markers generally associated with M2 phenotypes (TARC, Grob, PARC). OxLDLs therefore do not simply promote inflammation but disrupt the physiological orientation of macrophages in both directions.

Exposure to oxLDL after polarization (MΦ > oxLDL, Figure 9B) reprograms macrophages to a pro-inflammatory state. M0 and M2c are the two main populations affected, and oxLDL clearly turns their neutral or pro-resolutive phenotype to a more M1 phenotype associated with IL6, TNFa and IL-12p40 production. This transition is consistent with the role of oxLDL in the atherosclerotic microenvironment, where they contribute to the maintenance of a chronic inflammatory state. Conversely, oxLDL disrupts cytokine responses in M1 and M2d populations by reducing the levels of numerous markers—such as TNF, TARC, arginase, and MCP—thereby confirming previous observations regarding the disruption of macrophage polarization by oxLDL.

## 3. Discussion

This study aimed to characterize the effects of several major atherosclerosis-associated oxysterols across the different cellular compartments involved in atheromatous plaque development, namely, macrophages, vascular endothelial cells, and vascular smooth muscle cells. It is now recognized that oxysterols do not constitute a homogeneous group but fall into two broad classes with contrasting biological properties: enzymatically generated side-chain oxysterols (25-HC, 27-HC), which exert physiological immunometabolic functions primarily through liver X receptors (LXRs) [26,27], and non-enzymatically generated C7-oxidized oxysterols (7KC, 7β-HC), which are among the most biologically active oxysterols and can trigger diverse stress responses and cell death pathways depending on the cellular context [11,12,13,28,29].

This dichotomy frames the interpretation of our results. Whereas most studies focus on a single cell type, our data reveal strongly context-dependent responses, suggesting that oxysterols contribute to plaque progression through multiple complementary mechanisms. This comparative, multi-compartment approach addresses a recognized gap in the literature, as most mechanistic data have historically been generated in macrophages, even though smooth muscle cells are now acknowledged as a major source of foam cells [30,31,32]. Our data revealed distinct responses of macrophages, endothelial cells, and vascular smooth muscle cells to oxysterols, particularly regarding lipid uptake pathways, cholesterol handling, vascular endothelial dysfunction, oxidative stress, apoptosis, phagocytosis, and foam-cell formation. These findings emphasize that the impact of oxysterols on atherosclerosis extends beyond macrophages and involves distinct responses among vascular cell populations, underscoring the value of a multicellular approach to better reflect the complexity of the vascular wall environment.

Among the molecules studied, 7KC emerged as the oxysterol with the greatest biological activity. This observation is consistent with the literature describing 7KC as one of the most abundant oxysterols in oxidized LDL and in human atherosclerotic plaques, as well as a potent inducer of oxidative stress, inflammation, and cell death [33,34,35]. However, our findings reveal marked differences in the responses of macrophages, endothelial cells, and vascular smooth muscle cells to 7KC exposure, highlighting the cell-type-specific nature of oxysterol-induced vascular alterations.

One of the most notable observations concerns the distinction between neutral-lipid- and phospholipid-associated Nile Red fluorescence. In vascular cells, 7KC did not consistently promote neutral lipid accumulation. The neutral-lipid-associated signal remained close to control levels in carotid endothelial cells and was modestly increased in carotid vascular smooth muscle cells, whereas it was lower than control in abdominal endothelial and smooth muscle cells. By contrast, 7α-HC was associated with the highest neutral-lipid-associated signal in carotid endothelial cells, abdominal endothelial cells, and carotid vascular smooth muscle cells. In abdominal vascular smooth muscle cells, 27HC induced the largest increase in the neutral-lipid-associated signal. In addition, 7β-HC was associated with a decrease in neutral-lipid-associated fluorescence in abdominal endothelial cells. Overall, these descriptive trends identify 7α-HC and, in abdominal v-SMCs, 27HC, rather than 7KC, as the oxysterols that are most consistently associated with increased neutral lipid fluorescence in vascular models under the present experimental conditions.

The phospholipid-associated Nile Red signal showed a distinct, cell-type- and vascular territory-dependent pattern. In carotid endothelial cells, 7KC and 7α-HC were associated with the largest increases in phospholipid-associated fluorescence. In abdominal endothelial cells, this signal was predominantly increased by 27HC and 7α-HC, while 7KC remained close to control levels and 7β-HC was associated with a decrease. In carotid v-SMCs, 7α-HC and 7KC induced the most pronounced increases in phospholipid-associated fluorescence, whereas in abdominal v-SMCs, 7KC was the strongest inducer of this signal. These observations suggest that 7KC may preferentially affect phospholipid-associated Nile Red fluorescence in selected vascular cell models, particularly v-SMCs, rather than consistently promoting neutral lipid storage. Importantly, an increased phospholipid-associated Nile Red signal does not directly demonstrate increased cellular phospholipid content or foam-cell formation. It may reflect changes in membrane-rich intracellular compartments, membrane lipid organization, or the intracellular partitioning of the dye.

In macrophages, oxysterols elicited a response profile distinct from that observed in vascular cells. The neutral-lipid- and phospholipid-associated signal remained broadly comparable to control levels following exposure to these oxysterols.

These findings further indicate that the lipid-associated response to oxysterols is both compound- and cell-type-dependent.

Taken together, these results suggest that the mechanisms driving foam-cell formation differ across vascular compartments. In the present study, this process should be inferred primarily from changes in the neutral-lipid-associated Nile Red signal, whereas the phospholipid-associated signal provides complementary information on oxysterol-induced alterations in lipid-associated cellular compartments. In primary vascular cells, certain oxysterols may directly reprogram intracellular lipid metabolism independently of massive lipoprotein influx. This interpretation is particularly relevant considering work showing that v-SMCs account for at least 50% of the foam-cell population in advanced human and murine lesions, a contribution long underestimated by labeling strategies relying on macrophage markers alone [31,36,37]. It has further been established that v-SMCs can adopt a macrophage-like phenotype and become lipid-laden through oxLDL internalization, notably via LOX-1 [38,39,40]. Accounting for this quantitative contribution of v-SMCs therefore places vascular cells at the core of lipid-loading mechanisms, on par with macrophages.

In abdominal endothelial cells and v-SMCs, 7KC was associated with a lower neutral-lipid-associated Nile Red signal. In abdominal v-SMCs, this decrease occurred concomitantly with an increased phospholipid-associated signal, indicating that 7KC does not necessarily induce a global decrease in cellular lipid-associated fluorescence but may instead alter the distribution of Nile Red between neutral-lipid-rich and membrane-associated compartments. This pattern may reflect reduced neutral lipid droplet storage, enhanced lipid droplet turnover, and/or remodeling of membrane-rich intracellular compartments under 7KC-induced cellular stress. However, because phospholipid-associated Nile Red fluorescence does not directly quantify the cellular phospholipid content, these mechanisms remain speculative and require confirmation using orthogonal lipidomic, biochemical, or imaging approaches. The divergent responses between carotid and abdominal cells further support the existence of vascular-bed-specific differences in oxysterol handling and lipid metabolism. Moreover, in abdominal endothelial cells, 7β-HC decreased both neutral-lipid- and phospholipid-associated Nile Red fluorescence, suggesting a global alteration of lipid-associated cellular compartments rather than a selective reduction in neutral lipid storage. This profile may be related to 7β-HC-induced cellular stress and membrane remodeling but requires confirmation by complementary approaches.

Analysis of the proteins governing lipid homeostasis helps to further interpret the Nile Red findings and highlights marked differences between cell types and vascular territories. In macrophages, 7KC was associated with reduced CD36 and LOX-1 expression together with a marked activation of SREBP1- and NR1H4-related pathways, whereas neutral-lipid- and phospholipid-associated Nile Red fluorescence remained globally close to control levels. These observations suggest that macrophages primarily respond to 7KC through metabolic and transcriptional adaptation mechanisms rather than through substantial intracellular lipid accumulation. In contrast, vascular smooth muscle cells displayed a distinct response pattern.

In carotid v-SMCs, 7KC and, to a lesser extent, 7α-HC induced a marked increase in CD36 together with a reduction in ABCA1 and ABCG1 expression. This imbalance between lipid influx and cholesterol efflux is classically associated with foam-cell formation [41,42,43]. Interestingly, Nile Red spectral analysis revealed that this molecular signature was accompanied predominantly by increased phospholipid-associated fluorescence rather than neutral lipid accumulation consistent with enhanced lipid biosynthetic activity and membrane remodeling. By contrast, abdominal v-SMCs exhibited reduced neutral-lipid-associated fluorescence despite increased phospholipid-associated fluorescence, suggesting that oxysterol-induced activation of lipid metabolic pathways may lead to distinct intracellular lipid partitioning according to the vascular territory. These observations support the concept that carotid and smooth abdominal muscle cells differ in their handling of oxysterol-induced lipid stress.

Similarly, in endothelial cells, the oxysterols showing the strongest effects on neutral-lipid-associated fluorescence, particularly 7α-HC, were not necessarily those inducing the greatest deregulation of lipid transport markers. This dissociation suggests that endothelial lipid accumulation reflects not only cholesterol influx and efflux pathways but also broader changes in membrane organization and intracellular lipid trafficking.

Our data also reveal substantial heterogeneity of lipid homeostasis markers between vascular territories, as the responses of carotid and aortic cells are not superimposable. Thus, 7KC induces a marked increase in SREBP1 in carotid v-SMCs, whereas a decrease is observed in aortic v-SMCs, suggesting a lipid metabolism reprogramming specific to the carotid territory. This variability is consistent with the well-established concept of phenotypic heterogeneity of smooth muscle cells, which partly reflects their distinct embryonic origins along the arterial tree [44,45,46]. It also aligns data showing divergent expression profiles between atherosclerosis-prone and atherosclerosis-resistant vascular regions [47], as well as documented metabolic differences between the common carotid artery and the abdominal aorta [48,49]. Our observations reinforce this concept of regional specialization of the arterial tree and suggest that differential sensitivity to oxysterols may contribute to the uneven susceptibility of arterial territories to lesion development.

This variability is particularly evident for intracellular oxysterol accumulation and foam-cell formation. Vascular cells and macrophages display marked reductions in cell viability following exposure to 7KC, irrespective of the level of intracellular accumulation, which may be marked, low, or even undetectable in carotid endothelial cells and macrophages. These observations suggest that the biological consequences of oxysterol exposure depend less on absolute intracellular quantity than on each cell’s intrinsic capacity to metabolize, compartmentalize, or activate adaptive survival pathways, such as autophagy, described as a protective response of v-SMCs exposed to 7KC via Nox4/Atg4B [50], or ABCG1-mediated efflux, which protects macrophages from 7KC-induced apoptosis [51].

Overall, combining Nile Red spectral analysis with lipid homeostasis markers indicates that oxysterols do not uniformly trigger classical foam-cell formation. Rather, they differentially affect lipid uptake (CD36, LOX-1), cholesterol efflux (ABCA1, ABCG1), and lipogenic reprogramming (SREBP1), resulting in distinct patterns of neutral lipid storage and phospholipid-associated remodeling depending on both the vascular territory and the cell type considered.

The behavior of 27HC warrants particular attention. Unlike 7KC, it induces relatively few changes in lipid receptor expression, little reduction in cell viability, limited effects on macrophage functions, and weak oxidative stress, while nonetheless increasing the intracellular 7KC content. This dissociation between the absence of its own accumulation and the rise in intracellular 7KC is one of the most original findings of this study. As an endogenous LXR ligand in cholesterol-loaded cells [52], 27HC could reprogram intracellular cholesterol metabolism and indirectly modify the endogenous oxidative balance favoring non-enzymatic 7KC generation. Its ability to modulate gene expression through both LXR-dependent and LXR-independent mechanisms [53] is consistent with an upstream transcriptional action rather than direct toxicity. This hypothesis is consistent with the immunomodulatory role of 27HC, which promotes M2 polarization of human macrophages [54] and induces TNF-α production [55]. Thus, in contrast to 7KC, which acts as a strongly deleterious oxysterol, 27HC may contribute to plaque evolution through longer-term cellular regulatory mechanisms, illustrating the contrast between immunometabolic side-chain oxysterols and C7-oxidized oxysterols [28,29].

However, the observed intracellular accumulation of 7KC or 27HC warrants a more nuanced interpretation. In the present study, intracellular 27HC was assessed using an ELISA-based assay, whereas intracellular 7KC was evaluated by flow cytometry using a specific anti-7KC antibody and expressed as median fluorescence intensity (MFI). Although these approaches provide valuable comparative information regarding intracellular oxysterol-associated signals, neither constitutes an absolute quantification method. In contrast to GC-MS or LC-MS/MS, which are considered the gold-standard techniques for oxysterol quantification, ELISA and immunofluorescence-based measurements cannot be directly converted into intracellular sterol concentrations. Consequently, the present results should be interpreted as relative measurements reflecting the presence or changes in intracellular 27HC- and 7KC-associated signals rather than absolute intracellular oxysterol levels. Nevertheless, these approaches offer important practical advantages, including compatibility with the analysis of multiple cellular fractions for 27HC and single-cell resolution with simultaneous assessment of viability and phenotypic markers for 7KC. The observed discrepancies between intracellular oxysterol-associated signals and cellular viability further suggest that oxysterol-induced responses are not solely determined by intracellular accumulation but also involve cell-specific adaptive mechanisms, intracellular compartmentalization, and cholesterol-handling pathways. Future studies using targeted mass spectrometry would be valuable to confirm and extend these observations.

The study of endothelial cells confirms the central role of oxysterols in endothelial dysfunction, considered one of the initiating events of atherosclerosis [56,57]. 7KC induces a marked increase in ICAM-1 and E/P-selectin, a decrease in eNOS, a loss of EPCR, and a reduction in several angiogenic markers. Induction of adhesion molecules by 7KC through increased ROS production has been specifically documented in human aortic endothelial cells [58], and C7-oxidized oxysterols are known to induce differential profiles of IL-1β secretion, adhesion molecule expression, and endothelial apoptosis [59]. Together, these changes delineate a pro-inflammatory, pro-thrombotic, and dysfunctional endothelial phenotype liable to promote leukocyte recruitment, disruption of vascular homeostasis, and plaque instability. Notably, 7α-HC reproduces some of these effects very markedly at the carotid level despite a much lower impact on cell viability, suggesting that certain endothelial alterations represent a general response to oxysterols and are not solely a consequence of the cellular damage induced by 7KC. This observation is consistent with previous reports showing a dissociation between inflammatory signaling and alterations in cell viability for LXR-activating oxysterols acting through LXR-independent pathways [60]. Our endothelial cell findings are also consistent with previous studies reporting the deleterious effects of 7-oxysterols on vascular homeostasis. Notably, Li et al. [61] demonstrated that 7-ketocholesterol and related 7-oxysterols induce intracellular lipid accumulation, lysosomal dysfunction, and apoptosis in human endothelial cells, suggesting that disruption of intracellular lipid trafficking and lysosomal homeostasis contributes directly to endothelial injury. In agreement with these observations, we found that 7KC markedly reduced endothelial cell number, induced profound alterations in Nile Red-associated lipid signals, and promoted an endothelial dysfunction phenotype characterized by increased ICAM-1 expression together with reduced eNOS and EPCR expression. While the study by Li et al. [61] focused primarily on endothelial cells, our results extend these observations by demonstrating that the biological consequences of 7KC are highly dependent on both cell type and vascular territory. Furthermore, our spectral Nile Red analysis suggests that oxysterol-induced lipid remodeling may involve not only neutral lipid accumulation but also alterations in phospholipid-associated intracellular compartments, potentially reflecting broader disturbances in membrane organization and intracellular lipid handling pathways.

The results obtained in macrophages illustrate the complexity of oxysterol effects on innate immunity and show that these molecules do not merely modulate lipid loading but actively reprogram cellular polarization states. Within the classical paradigm, pro-inflammatory M1-type macrophages predominate in unstable, rupture-prone plaque regions, whereas alternatively activated M2-type macrophages are preferentially associated with stable plaques and inflammation resolution [62,63,64]. Our data fit within a framework in which oxidized lipids actively skew this balance toward a deleterious profile.

7KC exemplifies this imbalance. It induces strong ROS production, increased apoptosis, and reduced phagocytic capacity, a set of effects consistent with the well-described role of 7-oxocholesterol as a driver of M1/M2 imbalance toward a pro-inflammatory profile [62]. Moreover, 7KC-induced apoptosis in myeloid cells involves well-characterized mitochondrial and calcium-dependent pathways [56,65], consistent with the mitochondrial dysfunction and oxidative stress we observe. This profile contrasts with that of 27HC, whose immunomodulatory action is preferentially oriented toward M2 polarization [61] and TNF-α production [63], once again illustrating the functional divergence between C7-oxidized oxysterols and side-chain oxysterols.

In addition to the effects induced by individual oxysterols, oxidized LDL generated a distinct biological response profile, reflecting the complex composition of these particles.

In vascular cells, oxLDL exerted only minor effects on both neutral-lipid-associated (essentially a slight increase in neutral lipids within abdominal endothelial cells) and phospholipid-associated fluorescence relative to untreated controls. These observations do not support a strong oxLDL-induced neutral lipid accumulation in the vascular models under the present conditions. On the other hand, in macrophages, oxLDL markedly increased the neutral-lipid-associated Nile Red and the phospholipid-associated signals. Thus, although oxLDL affected both Nile Red fluorescence components, the prominent increase in the neutral-lipid-associated signal supports its capacity to promote macrophage neutral lipid loading and foam-cell formation. Indeed, previous studies have consistently shown that oxLDL promotes foam-cell formation through scavenger receptor-mediated uptake and intracellular cholesteryl ester accumulation [66]. In contrast with the macrophage response to oxLDL, exposure to purified 7KC induced only a limited increase in the neutral-lipid-associated signal in macrophages, despite its marked viability, pro-oxidant, and pro-apoptotic effects. This distinction indicates that lipid loading induced by oxLDL cannot be attributed solely to its 7KC content. Rather, it is likely to result from the uptake of the whole oxidized lipoprotein particle through scavenger-receptor-dependent pathways, together with the integrated effects of oxidized phospholipids, modified apolipoproteins, and cholesterol derivatives. Therefore, direct exposure to 7KC and indirect exposure through oxLDL trigger distinct macrophage responses: 7KC predominantly promotes cellular stress and impaired macrophage function, whereas oxLDL strongly promotes neutral lipid accumulation associated with foam-cell formation.

These profound changes in lipid homeostasis are not merely metabolic consequences but may also contribute to shaping macrophage functional responses. Accordingly, the macrophage phenotype induced by oxLDL appeared more complex than a simple M1 or M2 polarization state.

Indeed, the behavior of oxLDL is more complex and cannot be reduced to the binary M1/M2 scheme. Our observation of a hybrid activation state, combining features of alternatively activated and inflammatory macrophages, is in line with the description of the Mox phenotype induced by oxidized phospholipids through the Nrf2/HO-1 pathway [67,68]. It is also consistent with previous work showing that oxidized lipids confer pro-inflammatory properties on M2 macrophages [69,70,71].

The fact that oxLDL does not strictly reproduce the phenotype induced by 7KC alone suggests that oxidized lipoprotein particles deliver a composite signal combining oxysterols, oxidized phospholipids, and modified apolipoproteins, whose cellular integration differs from that of an isolated oxysterol [68,72].

Furthermore, our data indicate that oxLDL exposure before or after polarization profoundly alters the functional trajectory of macrophages. This dependence on timing suggests that oxLDL disrupts physiological processes of macrophage specialization and plasticity. Consequently, the impact of oxidized lipids may vary according to the maturation stage and pre-existing activation state of macrophages recruited into the lesion.

Collectively, these perturbations contribute to sustaining the chronic inflammatory microenvironment characteristic of the atherosclerotic plaque. Beyond inflammation, the reduced phagocytic capacity we observe carries particular significance: impaired efferocytosis is a recognized determinant of necrotic core expansion, fibrous cap thinning, and plaque vulnerability [73,74,75]. By simultaneously inducing macrophage apoptosis and reducing phagocytic capacity, 7KC may fuel a vicious cycle of accumulating apoptotic debris that promotes progression toward an unstable lesional phenotype.

Taken together, our results support the view that oxysterols should be regarded as a family of mediators with distinct biological properties. While 7KC emerges as the most potent inducer of oxidative stress, endothelial dysfunction, apoptosis and lipid homeostasis deregulation across vascular cell types, oxLDL remains the strongest driver of neutral lipid accumulation and classical foam-cell formation [33,35,76]. In contrast, 27HC and other side-chain oxysterols appear to exert predominantly regulatory effects on lipid metabolism and cellular functions, often associated with vascular-territory-specific patterns of lipid remodeling [26,52,54]. The integration of Nile Red spectral analysis with SREBP1, CD36, LOX-1, ABCA1 and ABCG1 expression further indicates that oxysterols alter not only the lipid content but also intracellular lipid partitioning and cholesterol handling pathways in a cell-specific manner. This diversity of action may partly explain the complexity of the mechanisms involved in plaque initiation, progression and destabilization.

The fact that 7KC exerts its effects across macrophages, endothelial cells, and smooth muscle cells suggests that it acts as a transversal mediator capable of affecting the entire vascular ecosystem involved in plaque progression. However, the present study also highlights that oxysterol-induced lipid alterations are not restricted to neutral lipid accumulation. Spectral Nile Red analysis revealed distinct patterns of phospholipid and neutral lipid remodeling depending on both the oxysterol and the cell type, indicating that oxysterol-induced dysfunction may involve broader disturbances of cellular lipid homeostasis and membrane organization. Nevertheless, 7KC is not the only active species: 7β-HC and the other oxysterols studied also modulate several cellular functions, corroborating the idea that the effects of oxidized LDL result from the combined, additive, or synergistic action of multiple oxidized cholesterol derivatives rather than the exclusive action of a single compound [28,34].

Finally, the predominance of 7KC reinforces the interest in strategies aimed at selectively neutralizing this oxysterol or limiting its biological consequences. Nevertheless, the non-negligible contribution of the other oxysterols argues for a broader approach capable of targeting several oxidized species simultaneously. In this context, cyclodextrins are of particular interest. Through their ability to sequester cholesterol and its derivatives within their hydrophobic cavity, they can act concomitantly on different oxysterols rather than on a single species. Thus, hydroxypropyl-β-cyclodextrin solubilizes and promotes the efflux of 7KC from foam-cell macrophages [76] and mobilizes free and esterified cholesterol from human foam cells [77]. At the integrated level, cyclodextrins promote atherosclerosis regression through macrophage reprogramming, enhanced cholesterol efflux, and LXR-dependent modulation of inflammation [78,79] while attenuating cholesterol crystal-induced inflammation through modulation of complement activation [80,81]. However, increased cholesterol efflux capacity does not systematically translate into lesion regression, as several experimental studies in ApoE−/− mice have reported dissociation between enhanced reverse cholesterol transport and atherosclerotic plaque reduction under specific conditions [82]. Beyond their capacity to bind and mobilize oxysterols, cyclodextrins have been widely reported to influence membrane cholesterol distribution and lipid raft organization. Consequently, the protective effects observed in the present study may arise not only from a reduction in oxysterol burden but also from modulation of membrane-associated signaling pathways involved in oxysterol sensing and cellular adaptation.

Collectively, these observations emphasize that oxysterol biology extends beyond foam-cell formation alone and involves complex alterations in lipid homeostasis, macrophage activation, and vascular-cell function. This complexity highlights the need for careful compound-specific evaluation of each cyclodextrin, considering both the chemical nature of its substituents and their degree of substitution, two parameters likely to govern sterol affinity, oxysterol sequestration capacity, and biological efficacy.

**Figure 10:**
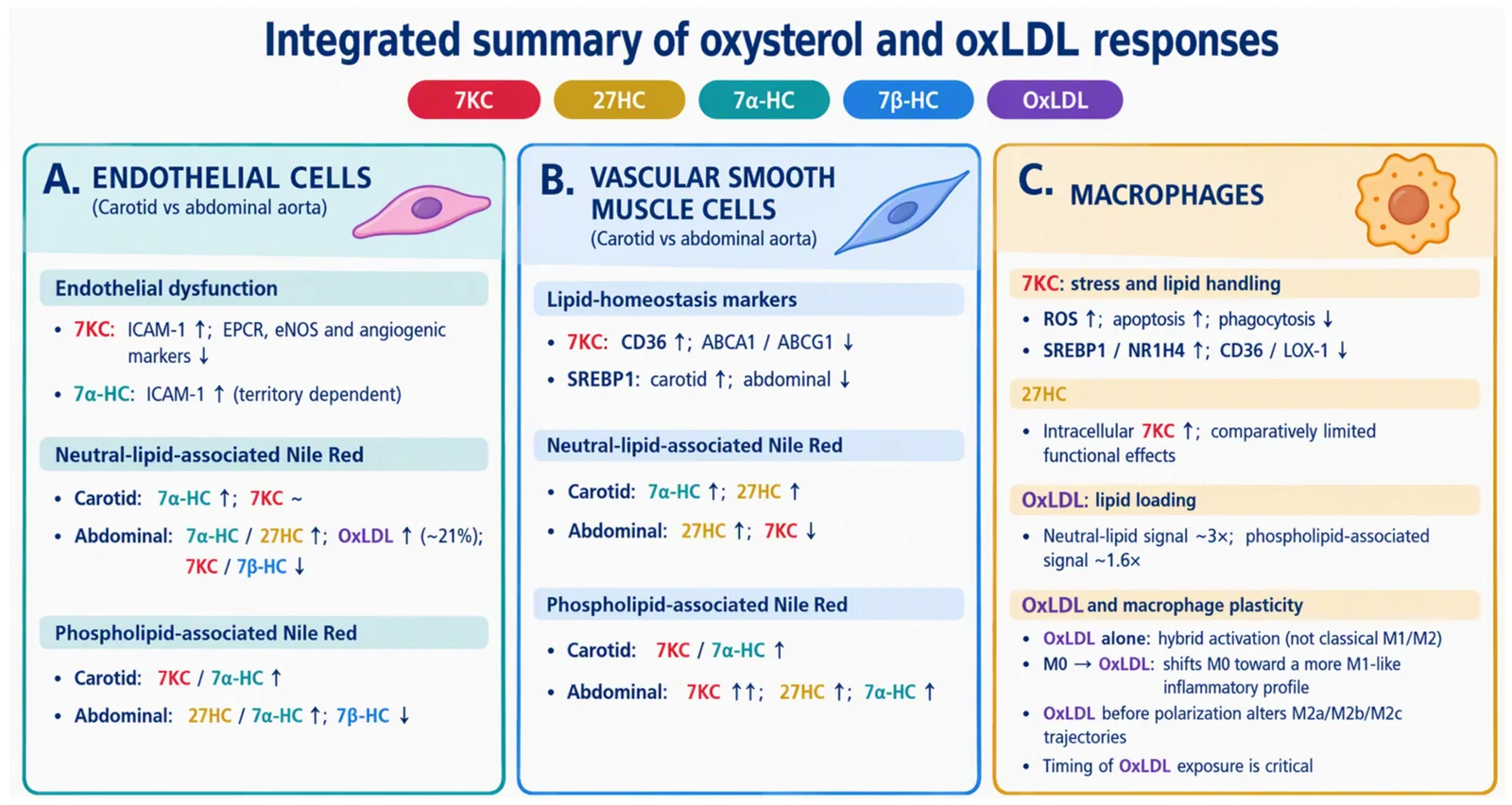
Integrated summary of cell-type- and vascular-territory-specific responses to oxysterols and oxidized LDL. Primary human vascular endothelial cells (v-ECs) and vascular smooth muscle cells (v-SMCs) isolated from the carotid artery or abdominal aorta, as well as human monocyte-derived macrophages, were exposed to selected oxysterols or oxidized LDL (oxLDL). Nile Red fluorescence is presented as separate neutral-lipid-associated and phospholipid-associated signals. (**A**) Endothelial cells. In endothelial cells, 7-ketocholesterol (7KC) increased ICAM-1 expression and decreased EPCR, eNOS, and several angiogenesis-associated markers. Lipid-associated responses differed between vascular territories. For the neutral-lipid-associated Nile Red signal, 7α-hydroxycholesterol (7α-HC) was associated with the highest increase in carotid v-ECs, whereas 7α-HC and 27-hydroxycholesterol (27HC) increased the signal in abdominal v-ECs. In abdominal v-ECs, 7KC and 7β-hydroxycholesterol (7β-HC) were associated with lower neutral-lipid-associated fluorescence. The phospholipid-associated signal increased predominantly with 7KC and 7α-HC in carotid v-ECs and with 27HC and 7α-HC in abdominal v-ECs; 7β-HC was associated with a decrease in abdominal v-ECs. (**B**) Vascular smooth muscle cells. In v-SMCs, 7KC increased CD36 expression and decreased ABCA1 and ABCG1 expression, whereas SREBP1 responses differed between carotid and abdominal cells. Neutral-lipid-associated Nile Red fluorescence was increased by 7α-HC and 27HC in carotid v-SMCs, while 27HC produced the largest increase in abdominal v-SMCs; 7KC was associated with lower neutral-lipid-associated fluorescence in abdominal v-SMCs. For the phospholipid-associated signal, 7KC and 7α-HC induced the strongest increases in carotid v-SMCs. In abdominal v-SMCs, 7KC was the strongest inducer, with increases also observed following 27HC and 7α-HC exposure. (**C**) Macrophages. In macrophages, 7KC increased reactive oxygen species (ROS), apoptosis, NR1H4, and SREBP1 expression while reducing phagocytic activity and CD36 and LOX-1 expression. In contrast, 27HC increased measured intracellular 7KC but had comparatively limited effects on the macrophage functions assessed. OxLDL markedly increased both the neutral-lipid-associated and phospholipid-associated Nile Red signals, with the most prominent increase observed for the neutral-lipid-associated signal, consistent with macrophage lipid loading. OxLDL also induced a distinct hybrid activation profile rather than a canonical M1 or M2 phenotype. Its effects on macrophage plasticity depended on the timing of exposure, including exposure of unpolarized M0 macrophages and exposure before or after polarization. Upward and downward arrows indicate increases and decreases relative to the corresponding untreated controls with statistical significance. The phospholipid-associated Nile Red signal is not a direct measurement of phospholipid mass or foam-cell formation. This schematic summarizes observed associations and does not imply that every compound was tested for every endpoint or that the pathways shown establish causality.

The main findings of this study are summarized in Figure 10, highlighting the cell-type- and vascular-territory-specific responses to oxysterols and oxLDL, as well as the timing-dependent effects of oxLDL on macrophage polarization.

## 4. Methods and Materials

### 4.1. Human Vascular Cell Collection and Culture

This study was conducted in accordance with the Declaration of Helsinki, and the collection of the tissues and associated research were approved by the Institutional Review Board and Ethics Committee of the French Ministry of Research (DC-2018-3186, obtained 14 September 2018) and the Agence de la Biomédecine under the agreement number PFS18-008 (obtained 7 June 2018). Fresh tissues (abdominal aorta and carotid artery) were collected in NaCl 0.9% (Cat. No. 3570380, B-Braun, Melsungen, Germany) supplemented with Penicillin/Streptomycin 1% (Cat. No. 509032, Clearline, Bernolsheim, France) at the French University Hospital of Nantes (CHU de NANTES), and cells were isolated within 24 h as previously described [73]. We washed the samples with cold PBS and then dissociated non-atheromatous vessels from organ donors with an enzymatic mix including collagenases (Liberase DL, Cat. No. 05401160001, Roche, Switzerland), elastase (porcine elastase, Cat. No. 07453, StemCells, Vancouver, BC, Canada) and DNAse (DNAseI from bovine, Cat. No. 10104159001, Roche, Basel, Switzerland) in high-glucose DMEM (Cat. No. 509051 Clearline, France). The first short digestion step permitted endothelial cell isolation. Then, mechanical dissociation followed by a longer incubation with digestion mix allowed smooth muscle cell isolation. For experiments, cells were used between passages 3 and 5. Vascular endothelial cells (v-ECs) and smooth muscle cells (v-SMCs) were isolated from the carotid artery and the abdominal aorta and were cultivated in complete medium (ECGM2 C-22011 and SMCGM C-22062, Promocell, Heidelberg, Germany) until day 5. Cells were then starved in deprivative basal medium supplemented with 2% FCS (Dutscher, Bernolsheim, France) without antibiotics during treatments.

### 4.2. Foam-Cell Formation and Detection

Vascular cells (endothelial and smooth muscle cells) were plated at a density of 8000-10000 cells/well in 96-well plates and cultivated 5 to 7 days in complete medium with one medium renewal. PBMC-MΦs were cultivated in RPMi (Cat. No. P04-17500, PanBiotech, Aidenbach, Germany) complete medium until day 7 with medium renewal at day 4. Cells were then incubated in deprivative medium (RPMI 2% human serum) supplemented with oxysterols at 40 µM (7KC: Cat. No. TN6715, 27HC: Cat. No. TQ0274, 7α-hydroxycholesterol (7a): Cat. No. T19161 and 7*β*-hydroxycholesterol (7b): Cat. No. TN1338, all purchased or prepared in DMSO at 10 mM, aliquoted and stored at −80 °C until used, TargetMol, Boston, MA, USA) or 30 µg/mL oxLDL (Cat. No. L34357, Thermofisher Scientific, Waltham, MA, USA) for an additional 48 h. Adherent cells were detached from the plate using Accutase (Corning, Corning, NY, USA), fixed in 2% paraformaldehyde (Cat. No. 28908, Thermo Scientific, Waltham, MA, USA) and stained with Nile Red (Cat. No. aat-22190, AAT bioquest, Pleasanton, CA, USA) for 30 min at room temperature. Neutral lipid signal (ex 488 nm/em 542 nm) and phospholipid signal (ex 488 nm/em 615 nm) were detected by spectral flow cytometry and analyzed with SpectroFlow v3.1 software (CYTEK, NLS, Fremont, CA, USA). Absolute cytometer cell counting (number of cells/µL) was used to address cell count for each condition.

### 4.3. Preparation of Human PBMCs and Macrophages

Healthy volunteers were recruited by the French Blood Bank Center (Etablissement Français du Sang, [EFS], Pays de Loire, Nantes, France, www.efs.sante.fr, accessed on 15 September 2026), which operates under the French Ministry of Health, and informed consent was obtained from all individuals. PBMCs from healthy donors were freshly isolated through Ficoll gradient separation (Cat. No. 17-1440-03, Ficoll plaque Plus, Cytiva, Marlborough, MA, USA). Monocytes were separated from PBMCs through adherence to plastic surfaces for 4 h in serum-free Roswell Park Memorial Institute (RPMI) 1640 (Cat. No. P04-17500, PanBiotech, Aidenbach, Germany) medium and were cultured in complete RPMI medium supplemented with 2% inactivated human serum (Cat. No. H3667, SIGMA Alfricd, Burlington, MA, USA), 1 mM non-essential amino acids, 1 mM pyruvate, and 10 mM 4-(2-hydroxyethyl)-1-piperazineethanesulfonic acid (HEPES) buffer (all purchased from LONZA, Basel, Switzerland), and 50 ng/mL macrophage colony-stimulating factor (M-CSF, Cat. No. 300-25, Peprotech, Cranbury, NJ, USA). Macrophage preparation was used for both Nile Red staining/toxicity studies and polarization assay between day 5 and day 8.

### 4.4. Polarization of PBMC-Derived Macrophages

Cells were treated with either 20 ng/mL IFN-γ (M1 macrophage differentiation inducer), 5 ng/mL IL-4 (M2a), 200 ng/mL poly I/C + 100 ng/mL LPS (M2b), 20 ng/mL IL-10 (M2c) or 50 ng/mL IL6 + 100 ng/mL LPS (M2d), from day 1 until day 8, with both medium and treatments being renewed on day 4. All cytokines were purchased from PeproTech, USA, poly I:C from R&D system USA and LPS from InvivoGen, San Diego, CA, USA. Oxysterol treatment (30 µg/mL oxLDL) was used preventively (from day 1 to day 4) before macrophage polarization or curatively (from day 4 to day 8) after macrophage polarization. The cell viability was assessed based on gating the NIR-Zombie-positive cells. The M0, M1, M2a, M2b, M2c and M2d macrophage populations were discriminated based on their expression of CD14/CD64/CD86/CD200R/CD206/CD163. Polygon plot view was performed using the Infinicyt software version 1.7.1e (Infinicyt, Cytognos, Salamanca, Spain). For the cytokine secretion assessment, the cells were stimulated with 100 ng/mL lipopolysaccharide (Cat. No. tlrl-b5lps, InvivoGen, USA) on day 7 and harvested on day 8. Supernatants (SNs) were collected after cell centrifugation, and the cytokine measurement was performed by ELISA (Cat. No. 741158 and 740503, LEGENDplex, BioLegend, San Diego, CA, USA) on frozen SNs. The secretion of the following cytokines was analyzed: ELC, BLC, MCP-2, IL-12p70, IL-12p40, TNF-α, IL-6, i-309, IL-23, IP-10, IFN-γ, IL-1β, IL-1RA, TARC, Arginase, MDC, MCP-4, IL-4, MCP-3, CX3CL1, Eotaxin-2, GRO-b, PARC, SDF-1 and IL-10.

### 4.5. Mitochondrial Reactive Oxygen Species (Mitoros) Assay

Freshly isolated PBMC-MΦs were cultivated in complete medium for 5 days and incubated with 40 µM oxysterols (7KC, 27HC, 7a and 7b) or 30 µg/mL oxLDL for an additional 24 h. MitoROS 580 (Cat. No. aat-16052, AATbioquest, Pleasanton, CA USA) was added to the medium for 30 min, adherent cells were detached from the plate using Accutase (Cat. No. 25-058-CI, Corning, USA), the cells were fixed in 2% paraformaldehyde (Cat. No. 28908, Thermo Scientific, USA), and the fluorescence signal (excitation 488 nm/detection 615 nm) was detected on a spectral flow cytometer and analyzed with SpectroFlow v3.1 software (CYTEK, NLS, Fremont, CA, USA).

### 4.6. Caspase 3/7 Apoptosis Assay

Freshly isolated PBMC-MΦs were cultivated in complete medium for 5 days and incubated with 40 µM oxysterols (7KC, 27HC, 7a and 7b) or 30 µg/mL oxLDL for an additional 72 h. Next, 2% DMSO (Cat. No. P60-36720100, Panbiotech, Aidenbach, Germany) was added to separate wells as a positive control for maximal apoptosis detection. The CellEvent Caspase-3/7 probe (Cat. No. C10423, Thermofisher Scientific, Waltham, MA, USA) was co-incubated with the compounds, and live cell imaging was performed at 37 °C until 48 h. One image was captured every 30 min using a GFP filter on an incubator microscope (Muvicyte, PerkinElmer, Waltham, MA, USA). Images were analyzed with Muvicyte Image Tools software version 2.0.18.

### 4.7. Phagocytosis Assay of PBMC-Derived Macrophages

Freshly isolated PBMC-MΦs were cultivated for 7 days in complete medium (M0) or polarized with IFN-γ (M1), IL-4 (M2a), poly I/C + LPS (M2b), IL-10 (M2c) or IL6 + LPS (M2d). OxLDL and 27HC were used as a source of oxysterol stress. At day 7, the cells were washed 2 times with pre-warmed RPMI phenol-free medium (Cat. No. P04-16516, Panbiotech, Aidenbach, Germany) and incubated with inactivated *E. coli* phRodogreen bioparticles (P35366, Invitrogen, USA) for 5 h on a live cell imaging system. One image was captured every 30 min using a GFP filter on an incubator microscope (Muvicyte, PerkinElmer, Waltham, MA, USA). Images were analyzed with Muvicyte Image Tools software version 2.0.18.

### 4.8. Flow Cytometry Analysis of Intracellular Lipid Receptors and Cell Surface Proteins

Cells (macrophages, endothelial cells and smooth muscle cells) were dissociated with soft enzymatic Accutase solution (Corning, USA), fixed in 2% paraformaldehyde (Cat. No. 28908, Thermo Scientific, Waltham, MA, USA), permeabilized in 0.1% Triton FACS buffer and stained for 30 min on ice for NR1H3, NR1H4, ABCA1, ABCG1, SREBP1, FASN, HMGCR, NOX2 (Biotechne, Minneapolis, MN, USA), 7KC (Thermofisher Scientific, Waltham, MA, USA), S1PR1, LDLR, CD36, LOX1, Arginase1 and eNOS (Biolegend, San Diego, CA, USA). Cell surface markers ICAM1, E/Pselectin, VCAM1, HLA-ABC, HLA-DR, HLA-E, CD39, Tie2, VEGFR2, CD146, CD31 (Biolegend, USA) and EPCR (Becton Dickinson, Franklin Lakes, NJ, USA) were detected following a 30 min incubation time on ice in conventional FACS buffer (PBS/2% FCS2/2 mM EDTA). The fluorescence signal was monitored on spectral flow cytometer and analyzed with SpectroFlow v3.1 software (CYTEK, NLS, Fremont, CA, USA).

### 4.9. Intracellular 27-Hydroxycholesterol (27HC) Detection

Freshly isolated PBMC-MΦs were seeded in 24-well plates (270.000 cells/well), cultivated in complete medium for 5 days and incubated for 48 h with 40 µM 27HC (Cat. No. TQ0274, targetMol, Linz, Austria) or 30 µg/mL oxLDL (Cat. No. L34357, Invitrogen, Carlsbad, CA, USA). The supernatants (SNs) were collected. After 2 washes with PBS, the isolation of membrane, cytoplasm and nucleus compartments was performed using a commercial cell fractionation kit (Cat. No. 42193S; Cell Signaling Technology, Danvers, MA, USA). Briefly, washed adherent cells were resuspended in 140 µL of Cytoplasmic isolation buffer and incubated for 5 min on ice. Supernatants were collected and labeled as Cytoplasmic fraction (C). The remaining cells were resuspended in 140 µL of Membrane isolation buffer and incubated for 5 min on ice. Supernatants were collected and labeled as membrane fraction (M). Finally, the remaining cells were incubated with 140 µL of Cytoskeletal and Nucleus isolation buffer, collected in Eppendorf tubes to be sonicated for 5 s and stored on ice. This fraction was labeled as Nucleus fraction (N). All fractions were measured immediately for 27HC expression using a commercial ELISA kit (Cat. No. MBS1608736; Mybiosource, San Diego, CA, USA) with a detection range from 2–720 ng/mL and a specificity of 1.3 ng/mL. The Optical Density Signal was acquired on a multiplate reader (Varioskan ALF, Thermofischer Scientific, Waltham, MA USA) and analyzed with SkanIt software version 7.1.0.41 RE (Thermofischer Scientific, Waltham, MA, USA).

### 4.10. Statistics

Variables are displayed as the mean ± SD. Data were analyzed by one- or two-way ANOVA followed by Dunnett’s multiple comparison test. The Mann–Whitney test was used as a nonparametric approach to compare intracellular 27HC levels to controls. Statistical analyses were performed using GraphPad Prism version 11.1.0(224) (GraphPad Software, Inc., La Jolla, CA, USA). A *p*-value < 0.05 was considered statistically significant. All in vitro experiments were performed in four to six individual donors with *n* = 3 technical replicates for PBMC-derived macrophages. A pool of four (v-SMCs) to eight donors (v-ECs) was used with *n* = 3 technical replicates for vascular cells.

## Data Availability

The data presented in this study are available on request from the corresponding author due to legal reasons.

## Supplementary Materials

The following supporting information can be downloaded at: https://www.mdpi.com/article/10.3390/ijms27219629/s1.
